# Unveiling the Mechanism of Plasma-Catalyzed Oxidation of Methane to C_2+_ Oxygenates over Cu/UiO-66-NH_2_

**DOI:** 10.1021/acscatal.4c00261

**Published:** 2024-05-02

**Authors:** Chong Qi, Yifu Bi, Yaolin Wang, Hong Yu, Yuanyu Tian, Peijie Zong, Qinhua Zhang, Haonan Zhang, Mingqing Wang, Tao Xing, Mingbo Wu, Xin Tu, Wenting Wu

**Affiliations:** † State Key Laboratory of Heavy Oil Processing, College of Chemical Engineering, Institute of New Energy, China University of Petroleum (East China), Qingdao 266580, P. R. China; ‡ Department of Electrical Engineering and Electronics, University of Liverpool, Liverpool L69 3GJ, U.K.; § National Engineering Research Center of Coal Gasification and Coal-Based Advanced Materials, ShanDong Energy Group CO., LTD, Jinan 250101, P. R. China; ∥ Sinopec Qingdao Refining & Chemical CO., LTD, Qingdao 266500, P. R. China

**Keywords:** plasma catalysis, CH_4_ oxidation, CO_2_ conversion, biogas reforming, alcohols, C_2+_ oxygenates

## Abstract

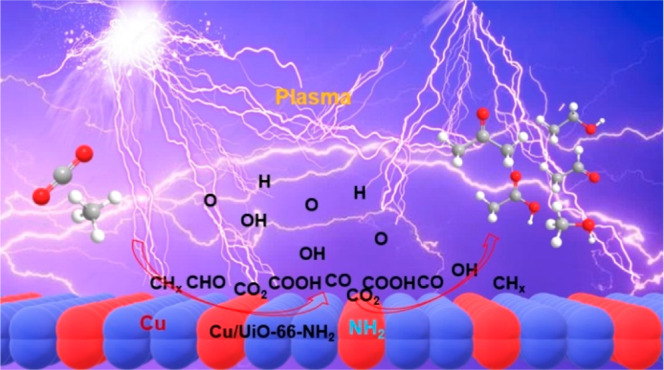

Nonthermal plasma (NTP) offers the potential for converting CH_4_ with CO_2_ into liquid products under mild conditions, but controlling liquid selectivity and manipulating intermediate species remain significant challenges. Here, we demonstrate the effectiveness of the Cu/UiO-66-NH_2_ catalyst in promising the conversion of CH_4_ and CO_2_ into oxygenates within a dielectric barrier discharge NTP reactor under ambient conditions. The 10% Cu/UiO-66-NH_2_ catalyst achieved an impressive 53.4% overall liquid selectivity, with C_2+_ oxygenates accounting for ∼60.8% of the total liquid products. In situ plasma-coupled Fourier-transform infrared spectroscopy (FTIR) suggests that Cu facilitates the cleavage of surface adsorbed COOH species (*COOH), generating *CO and enabling its migration to the surface of Cu particles. This surface-bound *CO then undergoes C–C coupling and hydrogenation, leading to ethanol production. Further analysis using CO diffuse reflection FTIR and ^1^H nuclear magnetic resonance spectroscopy indicates that in situ generated surface *CO is more effective than gas-phase CO (g) in promoting C–C coupling and C_2+_ liquid formation. This work provides valuable mechanistic insights into C–C coupling and C_2+_ liquid production during plasma-catalytic CO_2_ oxidation of CH_4_ under ambient conditions. These findings hold broader implications for the rational design of more efficient catalysts for this reaction, paving the way for advancements in sustainable fuel and chemical production.

## Introduction

The direct activation of methane (CH_4_) using carbon dioxide (CO_2_), a mild oxidant, under mild conditions presents a promising avenue for converting a range of CH_4_ sources, such as biogas generated from anaerobic digestion of organic waste or CH_4_ captured from coal mining operations, into valuable liquid products.^1−4^ This approach holds particular significance for remote locations where access to conventional gas or power grids is limited. However, the inherent stability of the C=O double bonds (5.5 eV) in CO_2_ and the C–H bonds (4.5 eV) in CH_4_ pose a significant challenge to direct conversion under conventional conditions. The strong bonds make it thermodynamically unfavorable to directly convert CH_4_ and CO_2_ to liquid products. As a result, an indirect two-step approach is typically employed, involving the energy-intensive dry reforming of methane (DRM) into syngas (a mixture of CO and H_2_) at high temperatures (>700 °C), followed by Fischer–Tropsch synthesis to produce liquid fuels and chemicals under high pressures.^5−8^ From an economic and energy-saving standpoint, direct conversion of CH_4_ and CO_2_ into liquid products under mild conditions is highly desirable, but it remains a significant challenge due to the thermodynamic limitations and the need for efficient catalysts that can facilitate the selective activation of these stable molecules.^9,10^

Due to its nonequilibrium characteristics, nonthermal plasma (NTP) emerges as a promising and attractive alternative for the activation of CH_4_ and CO_2_, enabling thermodynamically unfavorable reactions to take place under ambient conditions.^11,12^ High-energy electrons in the NTP collide with CH_4_ and CO_2_, inducing the decomposition of these gas molecules into reactive species, such as free radicals and excited species.^13−15^ These reactive species can then participate in various reactions, leading to the production of various products.^16−18^ While extensive research efforts have been dedicated to the plasma-catalytic DRM for the production of syngas,^19−21^ the direct conversion of CH_4_ and CO_2_ to oxygenates has received less attention. Recent work by Wang and co-workers demonstrated the substantial production of oxygenates from CH_4_ and CO_2_ using a water-cooled dielectric barrier discharge (DBD) plasma reactor under ambient conditions.^11,13^ The use of a Cu/Al_2_O_3_ catalyst enhanced the selectivity of acetic acid to 40.2%, while formaldehyde was only produced when using Pt/Al_2_O_3_ and Au/Al_2_O_3_.^11^ The relative importance of discharge power, reaction temperature, and residence time on reaction performance (e.g., conversion and selectivity) has also been investigated.^22^ Li et al. reported the plasma-catalytic conversion of CH_4_ and CO_2_ into oxygenates using Co/SiO_2_ and Fe/SiO_2_ catalysts.^23^ To date, a total oxygenate selectivity of 50–60% has been reported,^11^ with the production of acetic acid and methanol being the primary focus. Despite the progress made, the reaction mechanisms in the plasma-catalytic conversion of CH_4_ and CO_2_ into oxygenates remain poorly understood. Additionally, the synthesis of other C_2+_ oxygenates, such as ethanol, has remained less explored. This gap can be attributed to the initial activation of CH_4_ and CO_2_, where they are rapidly transformed into *COOH species that readily combine with *CH_3_/CH_3_ species to form acetic acid and methanol.^11,14^

Actually, the subsequent decomposition of *COOH into *CO and *OH, along with hydrogenation and C–C coupling processes, plays a crucial role in the plasma-catalytic conversion of CH_4_ and CO_2_. However, precise controlling these intermediate species and selective regulating the final product formation remains a challenge, especially in plasma catalysis.^24−28^ In conventional photo/electrocatalysis and thermocatalysis, base sites (e.g., –NH_2_ group) are used to adsorb and activate CO_2_, and Cu is among the preferred metal catalysts for stabilizing *COOH and *CO intermediates.^29,30^ More importantly, Cu-based catalysts demonstrate a remarkable electron-rich nature that facilitates multielectron transfer processes during CO_2_ reduction, significantly contributing to the effective conversion of CO_2_.^31,32^ Moreover, Cu has demonstrated a strong C–C coupling ability and can effectively promote the formation of C_2+_ liquid products.^33−38^ Due to their facile and designable functionality, metal–organic framework (MOF) materials (e.g., UiO-66) provide an excellent platform to combine the catalytic functions of the –NH_2_ group and Cu for the fabrication of model catalysts in plasma catalysis.^39^

In addition, most studies have focused on the role of original reactants/final products and catalysts by changing metal species and supports, while the formation of intermediate species during plasma catalysis remains largely unknown due to the limited in situ catalyst characterization methods available for plasma-catalytic chemical reactions. In situ plasma-coupled Fourier-transform infrared spectroscopy (FTIR) characterization has been developed recently, which could gain valuable insights into the plasma-assisted surface reactions and the possible reaction pathways.^12,40,41^ Despite this progress, the complex physicochemical interactions between plasma discharge and catalyst for efficient conversion of CH_4_ and CO_2_ into liquid products through plasma catalysis have not yet been attempted.

Here, we fabricated Cu-based NH_2_-decorated MOF catalysts for the one-step conversion of CH_4_ and CO_2_ into liquid products at room temperature and ambient pressure within a DBD reactor (Schemes S1 and S2). In situ plasma-coupled transmission FTIR was used to investigate the synergetic effect between –NH_2_ and Cu during the plasma-catalytic conversion process. Our findings reveal that –NH_2_ groups enhance the interaction between CH_4_ and CO_2_, facilitating the formation of key *COOH intermediates. The presence of Cu further promotes the transformation of these *COOH intermediates into *CO species, providing a foundation for subsequent hydrogenation and C–C coupling reactions. This synergistic interaction enables the 10% Cu/UiO-66-NH_2_ catalyst to achieve a remarkable 53.4% total liquid selectivity, with the highest reported selectivity for C_2+_ oxygenates. Notably, methanol (20.9% selectivity) is the major C_1_ product, followed by ethanol (18.4%) as the dominant C_2+_ oxygenates, with minor contributions from acetone (8.6%), acetic acid (3.3%), and acetaldehyde (2.3%).

## Experimental Section

### Chemicals and Reagents

ZrCl_4_, 1,4-benzenedicarboxylic acid, 2-amino-1,4-benzenedicarboxylic acid, *N*,*N*-dimethylformamide (DMF), acetic acid (CH_3_COOH), Cu(NO_3_)_2_·3H_2_O, and NaBH_4_ were purchased from Shanghai Aladdin Biochemical Technology Co., Ltd. All of the reagents were used without further purification.

### Preparation of UiO-66-NH_2_ and UiO-66

UiO-66-NH_2_ catalysts were prepared using a previously reported method.^29^ Typically, ZrCl_4_ (240.0 mg, 1.0 mmol) and 2-amino-1,4-benzenedicarboxylic acid (186.0 mg, 1.0 mmol) were each dissolved in 30 mL of DMF. The solutions were then mixed in a glass vial. After sonication for 10 min, deionized water (50 μL) and acetic acid (5.9 mL, 0.1 mol) were added to the mixture. Subsequently, the mixture was sealed and allowed to react at 120 °C for 24 h without stirring. The resulting products were collected by centrifugation and washed six times with DMF to remove unreacted precursors. Subsequently, the solvent was exchanged with acetone six times over a two-day period. The obtained UiO-66-NH_2_ was activated at 120 °C under a vacuum for 8 h. A similar process was used for the synthesis of UiO-66 using terephthalic acid (171.0 mg, 1.0 mmol).

### Preparation of *X*% Cu/UiO-66-NH_2_

The *X*% Cu/UiO-66-NH_2_ catalysts were prepared by using the incipient wetness impregnation method. First, 1.0 g of UiO-66-NH_2_ was dispersed in 200 mL of deionized water. Subsequently, 0.4 g of Cu(NO_3_)_2_·3H_2_O was added under stirring (10% Cu/UiO-66-NH_2_). The solution was stirred overnight at room temperature. After that, NaBH_4_ with 4 times the equivalent of copper was dissolved in water at 4 °C to prepare a fresh NaBH_4_ solution. This solution was then slowly dropped into the impregnated solution under vigorous stirring for 30 min. The solution immediately turned brown and then gradually turned black. After filtration, the obtained solid sample was washed repeatedly with deionized water and then dried at 60 °C in a vacuum oven for 8 h. Catalysts with different Cu loadings, namely, 15% Cu/UiO-66-NH_2_, 10% Cu/UiO-66-NH_2_, and 5% Cu/UiO-66-NH_2_, were obtained.

### Characterization of Catalysts

The X-ray diffraction (XRD) patterns of the samples were measured using a X’Pert PRO MPD (The Netherlands). X-ray photoelectron spectroscopy (XPS) analysis was performed using a Kratos AXIS Ultra spectrometer equipped with a prereduction chamber and an Elemental Vario EL III instrument (Elementar, Germany). Inductively coupled plasma atomic emission spectroscopy (ICP-AES) was conducted using a Shimadzu ICPS-8100 instrument. Scanning electron microscopy (SEM) images were recorded using a Hitachi s-4800 scanning electron microscope. Transmission electron microscopy (TEM) images were acquired by using a JEM-2100UH microscope operating at 200 kV. FEI Titan cubed Themis G2 300 Scanning TEM (STEM) with a spherical aberration corrector was used for high-resolution TEM (HRTEM) imaging. N_2_ physisorption measurements were carried out using a Micromeritics ASAP 2020 M microporous physical adsorption analyzer and a Tristar 3020 mesoporous physical adsorption analyzer. The samples (∼80 mg) were pretreated by degassing at 120 °C under a vacuum overnight. Thermogravimetric analyses (TGA) were performed using a NETZS 200F3/CHSTA449C instrument in the temperature range 50–600 °C at a heating rate of 10 °C/min. Differential thermogravimetry (DTG) represents the differential curve of the TGA.

CO_2_ physisorption measurements were performed by using a Micromeritics ASAP 2020 M microporous physical adsorption analyzer and a Tristar 3020 mesoporous physical adsorption analyzer. Samples were pretreated with vacuum degassing at 120 °C overnight. Then, the CO_2_ physisorption tests were conducted at 25 °C.

CO_2_ temperature-programmed desorption (CO_2_-TPD) was conducted using a Micromeritics AutoChem II 2920 instrument. The samples were first pretreated under flowing Ar (50 mL/min) at 180 °C for 1 h to remove any moisture. Afterward, the samples were cooled to room temperature to adsorb CO_2_ (1000 ppm of He at a flow rate of 50 mL/min) for 60 min. After CO_2_ adsorption, the system was purged with Ar (50 mL/min) for another 60 min to remove any physisorbed CO_2_ and gas phase CO_2_. The CO_2_-TPD measurements were then performed by ramping up the sample temperature from 50 to about 600 °C at a heating rate of 10 °C/min.

In situ diffuse reflectance infrared Fourier transform spectroscopy (DRIFTS) on CO adsorption was performed using a Thermo Nicolet NEXUS FTIR spectrometer. The catalyst was pretreated in an N_2_ atmosphere at 200 °C for 1 h and then cooled to 35 °C and injected with 5% CO/Ar mixture at a flow rate of 20 mL/min for CO adsorption. After 15 min of recording, pure N_2_ was injected for purge and desorption. Plasma-coupled in situ FTIR spectroscopy was performed in a self-designed transmission cell integrated with plasma for monitoring the plasma-derived surface reactions in this reaction. The experiment was conducted using a Jasco FT/IR-4600 FTIR spectrometer equipped with a Peltier stabilized DLaTGS detector with a resolution of 0.7 cm^–1^ using 32 scans. The experimental details are described in the Supporting Information.

### Experimental Setup

Scheme S1 shows the schematic diagram of the experimental setup. It comprises a high voltage AC power supply, a DBD plasma reactor, a product collection unit, and a product analysis unit. The DBD reactor comprises two concentric quartz tubes (Scheme S2). The inner quartz tube has a diameter of 15 mm, a thickness of 2.5 mm, and a length of 50 mm and is used as a dielectric material for the DBD reactor. Meanwhile, the outer quartz tube has a diameter of 15 mm, a thickness of 2.5 mm, and a length of 100 mm. A 316 stainless steel (SS) rod with an outer diameter of 14 mm functions as the inner high-voltage electrode and is coaxially positioned within the inner quartz tube. The inner quartz tube is sealed and insulated on both sides by the pressing plates made of polytetrafluoroethylene (PTFE). These conductive plates facilitate the disassembly and replacement of the catalysts. Unlike conventional DBD reactors, circulating water fills the gap between the two quartz tubes, serving as both the ground electrode and a coolant for the reactor. This design effectively maintains the reaction temperature at 20 °C by controlling the temperature of the circulating water, which is beneficial for the production of liquid products. The discharge gap is fixed at 3 mm, and the discharge volume of the reactor is fixed at 8 cm^3^. A high voltage probe (Tektronix P6015A) and a voltage probe were used to measure the voltages of the DBD reactor and external capacitor (100 nF). The electrical signals were collected by a digital oscilloscope (RIGOL DS1104z plus), and the discharge power was calculated using the Lissajous figure method.

### Plasma-Catalytic Experiments

In this study, the experiments were carried out at a discharge power of 10–40 W, a total flow rate of 50 mL/min (CH_4_/CO_2_ = 1:1 unless otherwise stated, without dilution), and a frequency of 8 kHz. 1.2 g of granulated catalysts (250–450 μm) was packed into half of the discharge region for each test (Scheme S2). A ceramic pad placed at the bottom of the reaction area supports the catalyst and facilitates the movement of reactant gases and products through the reactor. Prior to plasma reaction, the catalysts were subjected to a 20 min Ar pretreatment in the DBD reactor to clean their surfaces.

The gas products were measured in real time using a gas chromatograph (Scion 456C) equipped with a micropacked column (ShinCarbon ST), a thermal conductivity detector (TCD), and two flame ionization detectors (FIDs). Liquid products were collected in a cold trap containing 5 mL of deionized water. To maximize condensation of liquid products, the cold trap was placed in a large beaker with ice bags. The collected liquid products were introduced into the gas chromatograph via a headspace injector (DK5001a) and analyzed using a DB-WAX column and an FID. In addition, the gas volume before and after the reaction was measured using a soap-film flow meter at the outlet of the DBD reactor.

The liquid products were quantitatively analyzed by using H nuclear magnetic resonance (^1^H NMR) spectroscopy (Brook AV400). First, 10 μL of dimethyl sulfoxide (DMSO) was dissolved in 5 mL of deionized water and sonicated for 5 min. After that, 1 mL of the product was taken, and 50 μL of the mixture (H_2_O + DMSO) was added. The ultrasound was then performed for 5 min. Finally, 500 μL of the mixture (sample + DMSO + H_2_O) and 150 μL of deuterated water (D_2_O) were added.

### Analysis of Reaction Products

The chromatography was calibrated for each gaseous component by using standard calibration gases. The conversions (*X*) of CH_4_ and CO_2_ were defined as
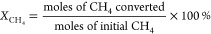
1
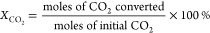
2

The selectivity (*S*) of gaseous products can be calculated
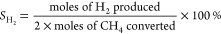
3
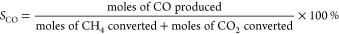
4
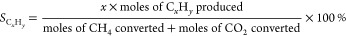
5

The chromatography was calibrated using a variety of standard liquid samples with different concentrations. The selectivity (*S*) of liquid products can be calculated according to the following equations
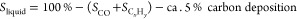
6
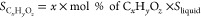
7
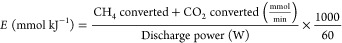
8

## Results and Discussion

### Structure Characterization

**Figure 1:**
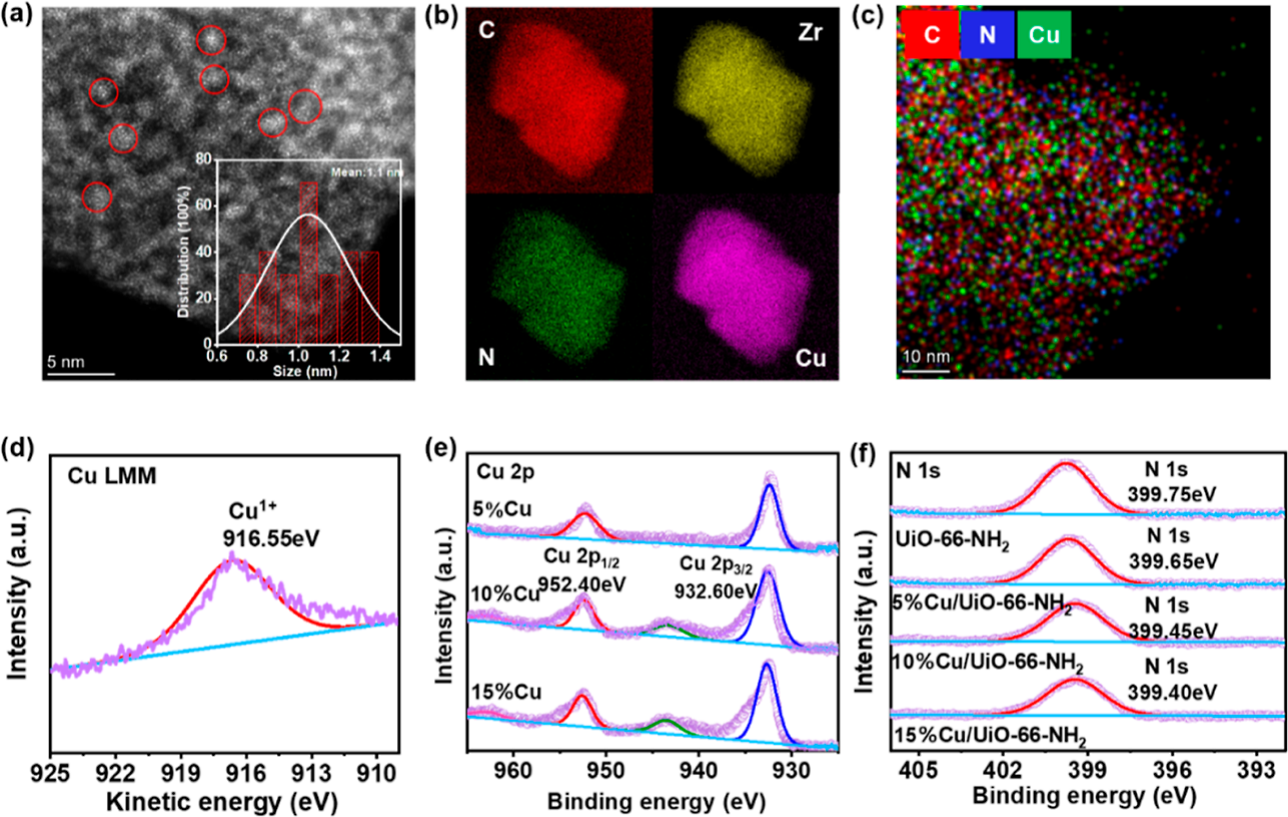
(a) STEM bright-field image and Cu particle diameter distribution (inset). (b) TEM mapping of 10% Cu/UiO-66-NH_2_. (c) STEM mapping of 10% Cu/UiO-66-NH_2_. (d) Auger Cu LMM spectrum of 10% Cu/UiO-66-NH_2_. (e) Cu 2p XPS profiles of *X*% Cu/UiO-66-NH_2_. (f) N 1s XPS profiles of UiO-66-NH_2_ and *X*% Cu/UiO-66-NH_2_.

We prepared UiO-66-NH_2_ supported Cu catalysts named *X*% Cu/UiO-66-NH_2_ (where *X* represents the Cu loading and *X* = 5, 10, and 15) using a facile impregnation method. According to ICP-OES results (Table S1), the Cu content of 5% Cu/UiO-66-NH_2_, 10% Cu/UiO-66-NH_2_, and 15% Cu/UiO-66-NH_2_ was 4.55, 8.95, and 13.23%, respectively. SEM and STEM images clearly showed that Cu is evenly distributed on UiO-66-NH_2_ (Figures S1 and 1a,b). The average particle size of the Cu species in 10% Cu/UiO-66-NH_2_ is about 1.1 nm (Figure 1a), and Cu is closely connected to N atoms (Figure 1c). As the Cu content increases, the specific surface area gradually decreases from 872 (UiO-66-NH_2_) to 603 m^2^/g (15% Cu/UiO-66-NH_2_), as shown in Figure S2 and Table S2, while the pore volume decreases from 0.36 (UiO-66-NH_2_) to 0.29 cm^3^/g (15% Cu/UiO-66-NH_2_). In contrast, the pore diameter increases from 3.42 (UiO-66-NH_2_) to 7.50 nm (15% Cu/UiO-66-NH_2_) with increasing Cu loading. A hysteresis loop is generated in the adsorption–desorption isotherms when the Cu content is increased to 15%, indicating that the excessive Cu species may destroy the micropores on the surface of the catalyst. Therefore, 10% Cu/UiO-66-NH_2_ with its proper Cu loading, could well preserve the original excellent properties of MOFs under NTP conditions.

The Cu species and their interaction with the –NH_2_ groups were further investigated. Auger Cu luminescence multiplexing microscopy (Cu LMM) spectra of 10% Cu/UiO-66-NH_2_ showed a peak at 916.55 eV, indicating the presence of Cu^+^ (Figure 1d).^42^ Associated with two sharp diffraction peaks at 36.4 and 42.3° in the XRD pattern, the Cu species could be attributed to Cu_2_O (Figure S3).^43^ As the Cu loading increased, the –NH_2_ peaks attributed to the shear vibration red-shifted gradually from 1620 to 1650 cm^–1^ and 1660 and 1670 cm^–1^ in the FTIR spectra (Figure S4a,b).^44^ These findings collectively suggest the presence of a discernible interaction between Cu and the amino group within the catalyst. Additionally, in the XPS analysis, the Cu 2p characteristic peaks showed a positive shift (Figure 1e), and the N 1s exhibited a negative shift (Figure 1f). These shifts indicate that the introduction of Cu enhanced the electron density of the –NH_2_ group, thereby further confirming the existence of a strong interaction between Cu and the –NH_2_ group (Figure S5). CO_2_-TPD analysis revealed that UiO-66-NH_2_ and 10% Cu/UiO-66-NH_2_ processed more weak and medium basic sites than those of UiO-66 (Figure S6a).^45,46^ More importantly, the CO_2_ adsorption capacity of 10% Cu/UiO-66-NH_2_ at room temperature was stronger than that of UiO-66-NH_2_ (Figure S6b), even though the specific surface area of 10% Cu/UiO-66-NH_2_ was smaller than that of UiO-66-NH_2_ (872 m^2^/g). These findings indicate that the presence of Cu may be beneficial for CO_2_ conversion.

### Plasma-Catalytic Conversion of CH_4_ and CO_2_

**Figure 2:**
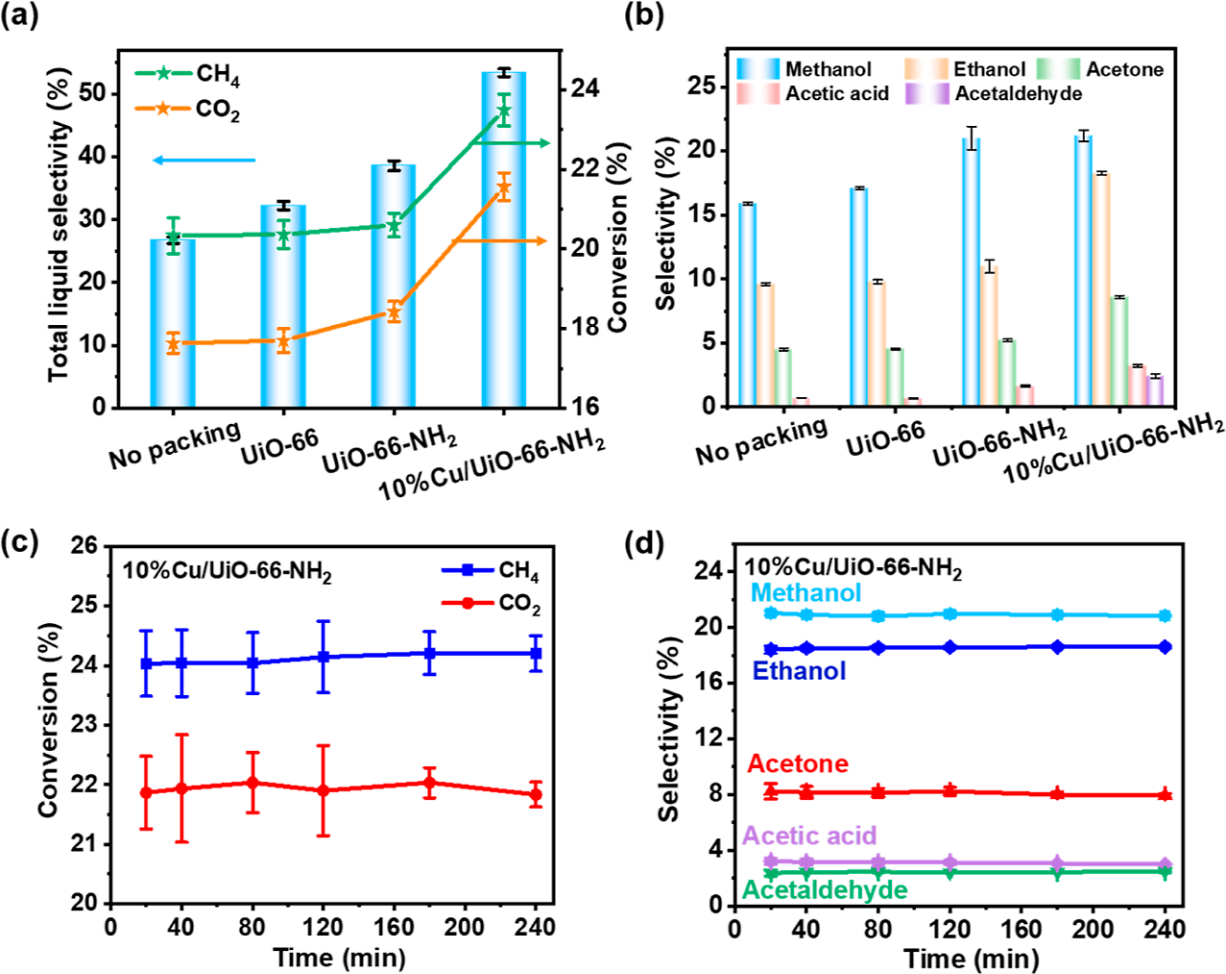
(a) Gas conversion and total liquid selectivity. (b) Selectivity of the main liquid products. Catalyst stability using 10% Cu/UiO-66-NH_2_ (c) gas conversion and (d) selectivity of main liquid products over time on stream (total flow rate = 50 mL/min, CH_4_/CO_2_ = 1:1, discharge power = 20 W).

The effect of the discharge power on the plasma-catalyzed conversion of CH_4_ and CO_2_ was first investigated (Figure S7). To ensure optimal conversion of CH_4_ and CO_2_ while maintaining liquid selectivity, the discharge power was fixed at 20 W (Figure S7). When the DBD reactor was packed with UiO-66 (Figure 2a), the conversions of CH_4_ (20.4%) and CO_2_ (17.7%) were similar to those without packing (CH_4_: 20.3% and CO_2_: 17.6%). When catalyzed by amino-functionalized UiO-66-NH_2_, it showed similar CH_4_ conversion but slightly enhanced the CO_2_ conversion. Additionally, the selectivity of liquid products reached 38.6%, higher than that of no packing (30.7%) and UiO-66 (32.2%). These findings suggest that –NH_2_ groups contribute to CO_2_ conversion to some extent on the catalyst surface. The CO_2_-TPD results (Figure S6) reveal a significant increase in both weak and medium basic sites on the UiO-66-NH_2_ catalyst compared to those of the pristine UiO-66. These additional basic sites enhance CO_2_ adsorption, facilitating its interaction with high-energy electrons and ultimately leading to a higher level of CO_2_ conversion.

Interestingly, the catalytic performance was significantly improved by the introduction of Cu. Among the catalysts with various Cu loadings, 10% Cu/UiO-66-NH_2_ showed the best catalytic performance (Figure S8), wherein CH_4_ and CO_2_ conversion increased to 23.5 and 21.6%, respectively. Importantly, the presence of Cu greatly improved the overall liquid selectivity to 53.4%, an increase of 74.0% compared to no packing. At the same time, the selectivity of H_2_ and CO substantially decreased to 32.0 and 26.5%, respectively (Figure S8b). All experimental and calculation results are provided in Tables S3–S8.

For the liquid product distributions, methanol and ethanol were the main products, followed by acetone, acetic acid, and acetaldehyde (Figure 2b). The liquid product distributions of UiO-66 were similar to those of no packing. The alcohol selectivities catalyzed by UiO-66-NH_2_ (methanol 21% and ethanol 10.9%) were higher than those of UiO-66, indicating that the introduction of the –NH_2_ group could promote the reaction on the surface of the catalysts. After the introduction of Cu, the superior C–C coupling ability of Cu resulted in a total selectivity of 32.5% for C_2+_ oxygenates, which was 113.8% higher than that using UiO-66 (Table S3). Among the C_2+_ liquid products, ethanol selectivity showed a significant increase of 84% compared to that of UiO-66. Similarly, acetone and acetic acid exhibited a 1.3-fold and 4.8-fold increase, respectively, compared to UiO-66 and no packing. The catalytic performance of 10% Cu/UiO-66-NH_2_ remained stable even when the reaction time was prolonged to 240 min (Figure 2c,d). The CH_4_ conversion could be consistently maintained at ∼23.5%, while the CO_2_ conversion could be stabilized at ∼21.6%. It is worth noting that the selectivity of primary liquid products remained highly stable. The decomposition of carbon deposit polymers was carefully analyzed using TGA (Figure S9). Various characterizations also confirmed the stability of the catalyst structure under plasma conditions (Figures S10 and S11).

### Reaction Mechanisms

**Figure 3:**
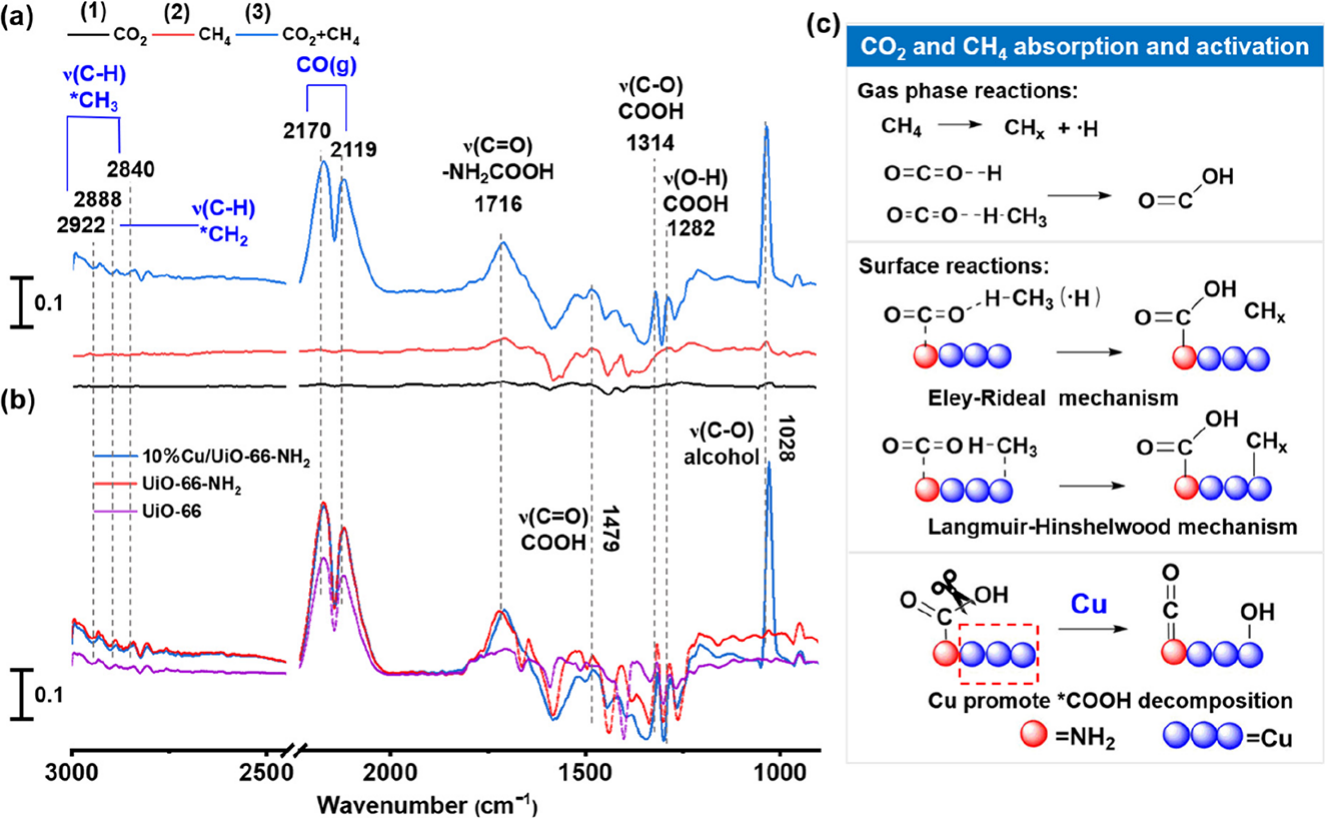
In situ transmission FTIR spectra of (a) DBD (discharge power = 15 W) under different atmospheres with continuous flow (10% Cu/UiO-66-NH_2_). (b) DBD packed with different catalysts at 15 min (CO_2_/CH_4_/Ar = 1:1:2, discharge power = 15 W). (c) Proposed mechanism for CO_2_ and CH_4_ absorption and activation, and the role of Cu in promoting *COOH conversion.·.

To further understand the plasma-assisted surface reaction and verify the proposed hypothesis, we performed in situ transmission FTIR characterization under plasma conditions (Figure 3). First, we investigated in situ plasma-coupled FTIR characterization under different atmospheres to elucidate the main reaction pathways (Figures 3a, S12–S14 and Table S9).

#### Condition (1)

Pure CO_2_ atmosphere (Figure 3a (1) and Table S10). For UiO-66, weak *COOH peaks were observed at 1496, 1314, and 1282 cm^–1^.^47,48^ Previous studies reported^49^ that CO_2_ could bind to the *OH group on the surface of UiO-66 to form *COOH. For UiO-66-NH_2_, the higher peak intensity of *COOH suggests that the introduction of –NH_2_ enhanced the chemsorption of CO_2_. This is because the alkaline nature of the –NH_2_ group facilitates the chemisorption of CO_2_ to form –NH_2_COOH species, which can be confirmed by the detection of carbamic acid species with the C=O stretching vibrations at 1716 cm^–1^. Note that the infrared peaks (1282, 1314, and 1475 cm^–1^) associated with the *COOH species on Cu/UiO-66-NH_2_ are weaker than those of UiO-66-NH_2_, indicating that the introduction of Cu promoted the further transformation of the generated *COOH. In addition, the presence of a single IR peak at 1028 cm^–1^ suggests the formation of alcohols through further hydrogenation of *COOH on the Cu particle surface. This is likely due to Cu sites promoting the decomposition of *COOH into *CO (R1), followed by the stepwise hydrogenation of *CO with limited surface *H to produce alcohols on the surface.

#### Condition (2)

The catalyst surface was initially adsorbed with CO_2_, followed by a reaction in a CH_4_ atmosphere (Figure 3a (2)). Under this condition, more obvious peaks attributed to *COOH (1479, 1314, and 1282 cm^–1^) could be observed, further confirming that the existing reaction pathway involved CO_2_ hydrogenation (*H/H + *CO_2_ → *COOH). The source of hydrogen may be from the *H from *CH_4_ (R2) or H radicals produced by CH_4_ decomposition under plasma conditions. In addition, the IR peak (1028 cm^–1^) associated with the formation of alcohols was significantly enhanced due to the increased concentration of both gas-phase and surface H and CH_3_ species when CH_4_ was present under plasma conditions in the system. Methanol, a major product, can be formed through both CO hydrogenation (R3) and the reaction between adsorbed *OH with gas-phase or surface CH_3_ species (R4). Additionally, C-C coupling processes can occur between adsorbed *CO and gaseous/surface CH_*x*_ (*x* = 1, 2, 3) species to generate ethanol (R5). Ethanol can also be formed through the combination of *CH_2_OH and *CH_3_/CH_3_ via both Langmuir–Hinshelwood (L–H) and Eley–Rideal (E–R) mechanisms.

#### Condition (3)

The reaction was performed under an atmosphere of CO_2_ and CH_4_ with a CH_4_/CO_2_ ratio of 1:1 (Figure 3a (3)). Under this condition, two characteristic absorption peaks associated with gaseous CO (CO (g) (2170 and 2119 cm^–1^) could be observed. More importantly, the peak corresponding to *COOH was further strengthened, indicating that there was sufficient H binding to CO_2_ probably on the catalyst surface or in the gas phase (Figure 3c). At the same time, the intensity of IR peaks corresponding to *CH_*x*_ (*x* = 2, 3) species also showed a significant increase, indicating an enhancement of reaction R2.^50^ The simultaneous rise in surface *COOH and *CH_*x*_ suggests a higher potential for surface reactions and strengthens the possibility of the L–H mechanism being dominant. Additionally, the significant enhancement of IR peaks for alcohols implies a substantial increase in surface species on the 10% Cu/UiO-66-NH_2_ catalyst due to the promoted L–H mechanism.

To understand the role of the –NH_2_ group in the plasma-catalytic conversion of CO_2_ and CH_4_, in situ transmission FTIR was performed on various catalysts (UiO-66, UiO-66-NH_2_, and 10% Cu/UiO-66-NH_2_) under condition (3) (Figures 3b and S15, and S16). Compared to UiO-66, UiO-66-NH_2_ and 10% Cu/UiO-66-NH_2_ showed higher intensities for the C–H stretching vibration peaks of *CH_3_ at 2888 and 2840 cm^–1^, as well as for the C–H stretching vibration peak of *CH_2_ at 2922 cm^–1^. Additionally, the *COOH peaks (1496, 1314, and 1282 cm^–1^) were significantly weaker in UiO-66 compared to UiO-66-NH_2_. Weak peaks associated with *CH_3_ and *CH_2_ were observed in UiO-66-NH_2_ and 10% Cu/UiO-66-NH_2_ under condition (2) (Figure S12b). However, under condition (3), the intensity of peaks at 2888, 2840, and 2922 cm^–1^ increased significantly. These results suggest that the strong interactions between CO_2_ and CH_4_ promote their conversion (Figures 3c and S12c).

**Figure 4:**
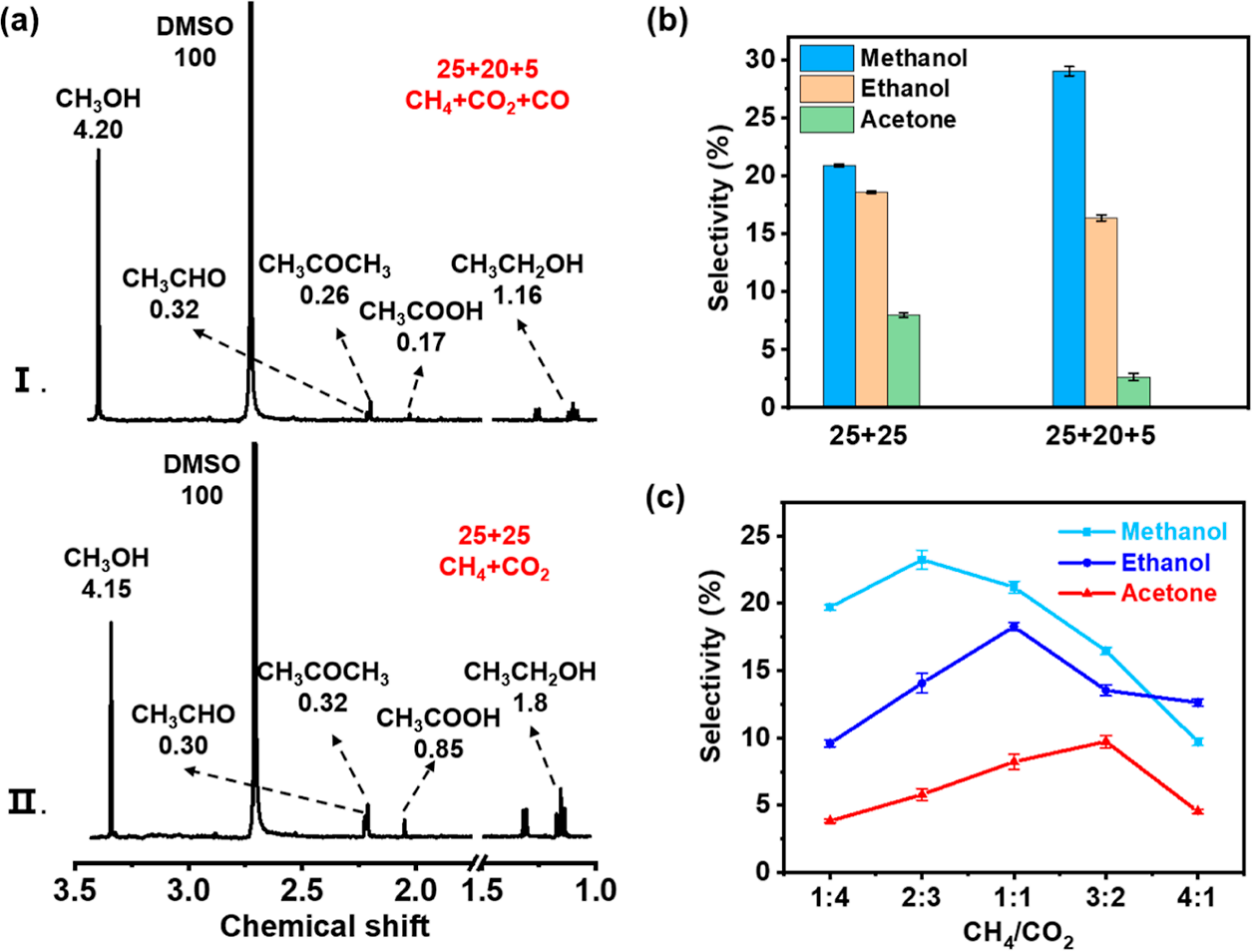
(a) ^1^H NMR spectra of major liquid products, with number indicating the integration of H atoms for each liquid product. (b) Effect of CO addition on the selectivity of main liquid products using 10% Cu/UiO-66-NH_2_ (total flow rate = 50 mL/min, CH_4_/CO_2_ = 25:25 (left), CH_4_/CO_2_/CO = 25:20:5 (right), discharge power = 20 W). (c) Selectivity of the main liquid products using 10% Cu/UiO-66-NH_2_ at different CH_4_/CO_2_ ratios (total flow rate = 50 mL/min, discharge power = 20 W).

In addition, the peak intensities at 1282 cm^–1^ (O–H bending vibration) and 1314 cm^–1^ (C–O vibration) were weaker for 10% Cu/UiO-66-NH_2_ compared to UiO-66-NH_2_ and UiO-66 (Figure 4b). This finding indicates that the presence of Cu particles promotes the decomposition of *COOH into *CO and *OH.^33^ Notably, the peak intensity for alcohols (1028 cm^–1^) was significantly stronger for 10% Cu/UiO-66-NH compared to the other catalysts (Figure 4a), suggesting that Cu plays an important role in promoting the formation of alcohols. In addition to the gradual hydrogenation pathway of *COOH, the combination of *OH and *CH_3_ can also enhance alcohol production.^51^

Interestingly, when catalyzed by 10% Cu/UiO-66-NH_2_, the selectivity of C_2+_ oxygenates such as ethanol reached 18.4%, which was higher than that obtained using UiO-66-NH_2_. This finding indicates that Cu promoted the coupling of C–C bonds. Among the various intermediates, *CO is an important intermediate species for C–C coupling. To prove the influence of Cu on *CO, in situ CO-DRIFTS was performed on UiO-66-NH_2_ and 10% Cu/UiO-66-NH_2_ (Figures S17 and S18). By comparison, it was found that only 10% Cu/UiO-66-NH_2_ had chemical adsorption capacity for CO (Supporting Information for detailed analysis), which benefits the migration of *CO from –NH_2_ to Cu sites.

To further understand the role of the important intermediates (CO (g) and *CO) in the plasma-catalytic system, without affecting the discharge, we conducted comparative experiments by adding a small amount of CO (CH_4_/CO_2_/CO = 25:20:5) to the original plasma-catalytic reaction (CH_4_/CO_2_ = 25:25), and then used ^1^H NMR to detect the changes of C_2+_ oxygenates (Figure 4a). When CO was added, the selectivity of ethanol and acetone was reduced to 16.1 and 2.6%, respectively. Compared with the gas phase CO (g), in situ generated *CO is more conducive to C–C coupling reactions on the catalyst surface. This leads to a higher yield of liquid products containing C_2+_ (Figure 4b), primarily through the L–H mechanism. Moreover, the selectivity of methanol increased to 29.5% with the addition of CO. This indicates that the gas-phase CO species do not readily couple with surface-bound methyl groups (*CH_3_), resulting in an enhanced reaction between *CH_3_ and hydroxyl groups (*OH) to form methanol. Meanwhile, adsorbed *CO species facilitate the surface-mediated C–C coupling reactions, leading to the formation of ethanol and acetaldehyde following the hydrogenation process (R5 and R6).
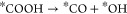
R1
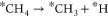
R2
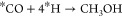
R3
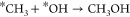
R4
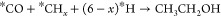
R5

To gain further insights into the influence of the CH_4_/CO_2_ ratio on the types of liquid products formed, we conducted experiments using 10% Cu-UiO-66-NH_2_ at various CH_4_/CO_2_ ratios (Figures 4c, S19 and Table S10). At a CH_4_/CO_2_ ratio of 2:3, methanol selectivity peaked, and no formic acid or formaldehyde was observed. This finding suggests that the formation of *OH can be enhanced under hydrogen-deficient reaction conditions. At a CH_4_/CO_2_ ratio of 1:1, ethanol reached its maximum value, while acetaldehyde was at its minimum, indicating that acetaldehyde could further transform into ethanol through hydrogenation with an appropriate CH_4_/CO_2_ ratio (R7). Acetic acid also reached its maximum at a CH_4_/CO_2_ ratio of 1:1, indicating that more active oxygen from CO_2_ was required for its formation. Here, the direct coupling of *COOH with *CH_3_/CH_3_ species forms acetic acid (R8 and R9). At a CH_4_/CO_2_ ratio of 3:2, acetone production peaked in this reduction atmosphere due to the combination between CH_3_ species and *CO (R10 and R11), rather than propane oxidation. As the CH_4_/CO_2_ ratio increased to 4:1, acetone production decreased, possibly due to reduced amounts of *CO.
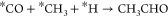
R6
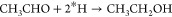
R7
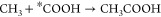
R8
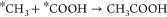
R9
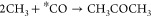
R10
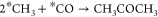
R11

**Scheme 1:**
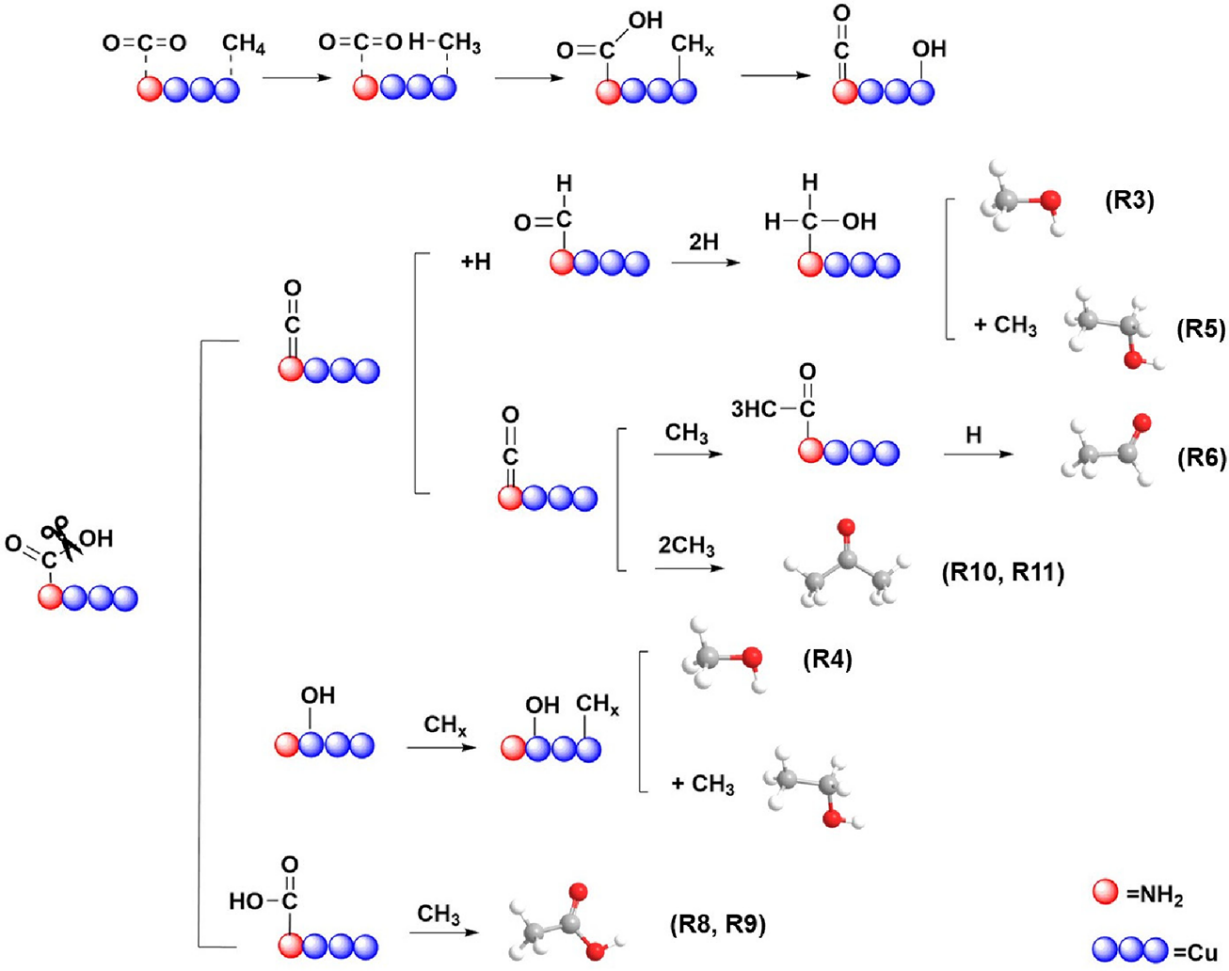
Possible Reaction Pathways of Effective Synergy between Plasma and Catalyst

Scheme 1 proposes a possible reaction pathway for the effective synergy of plasma on the surface of 10% Cu/UiO-66-NH_2_. Under plasma conditions, the –NH_2_ group (a strong Lewis base site) of the catalyst facilitates CO_2_ adsorption and its subsequent activation into *CO and *COOH. The introduction of Cu promotes the decomposition of *COOH to *CO, which is more conductive to the subsequent hydrogenation and C–C coupling that produce alcohols, aldehydes, and acetone. In addition, the highly reactive *OH species generated from *COOH can react with *CH_*x*_ species to produce more *CH_*x*_O species, which could further form C_2+_ liquid products through C–C coupling on Cu sites, thereby suppressing the formation of gaseous products (such as CO and H_2_).

## Conclusions

In this work, we designed co-functionalized catalysts incorporating Cu and –NH_2_ groups for the conversion of CH_4_ with CO_2_ into liquid products at room temperature and atmospheric pressure in a DBD plasma reactor. The synergy between Cu and the –NH_2_ group facilitates the decomposition of *COOH into *CO and *OH intermediates, stepwise hydrogenation, and C–C coupling reactions, leading to a controlled distribution of liquid products. With the 10% Cu/UiO-66-NH_2_ catalyst, we achieved an impressive 53.4% overall liquid selectivity, with C_2+_ liquid products accounting for ∼60.8% of the total liquid products. Notably, the distribution of C_2+_ oxygenates was shifted toward ethanol, outperforming acetic acid, which was previously reported as the dominant oxygenate. This work represents a significant breakthrough in the direct activation of CH_4_ with CO_2_ for the synthesis of C_2+_ oxygenates by using plasma catalysis under ambient conditions. This research highlights the transformative potential of plasma electrification processes for renewable-powered, decentralized production of fuels and chemicals, opening doors for a more sustainable future.

## Supporting Information Available

In situ FTIR characterization of the catalyst surface under plasma discharge, schematic diagram of experimental setup and plasma reactor, supplementary characterization of fresh catalysts, supplementary reaction performance, characterization of spent catalysts, in situ plasma-coupled FTIR characterization results, in situ CO-DRIFTS characterization results, and total liquid selectivity using 10% Cu/UiO-66-NH_2_ at different CH_4_/CO_2_ ratios (PDF)
