# Supplementary material for: Unveiling the Mechanism of Plasma-Catalyzed Oxidation of Methane to C2+ Oxygenates over Cu/UiO-66-NH2
Source: ACS Catal. 2024 May 2;14(10):7707–16. doi: 10.1021/acscatal.4c00261 (PMC11106747; doi:10.1021/acscatal.4c00261)
Supplement: Supplementary file 1 — cs4c00261_si_001.pdf [file cs4c00261_si_001.pdf]

Supporting Information

Unveiling the Mechanism of Plasma-catalyzed Oxidation of Methane to C_{2+} Oxygenates over Cu/UiO-66-NH_2

Chong Qi[1†], Yifu Bi[1,4†], Yaolin Wang[2†], Hong Yu[1], Yuanyu Tian[1], Peijie Zong[1], Qinhua Zhang[1], Haonan Zhang[1], Mingqing Wang[3], Tao Xing[3], Mingbo Wu[1,*], Xin Tu[2,*], Wenting Wu[1,*]

[1] State Key Laboratory of Heavy Oil Processing, College of Chemical Engineering, Institute of New Energy, China University of Petroleum (East China), Qingdao 266580, P. R. China

[2] Department of Electrical Engineering and Electronics, University of Liverpool, Liverpool L69 3GJ, U.K.

[3] National Engineering Research Center of Coal Gasification and Coal-Based Advanced Materials, ShanDong Energy Group CO., LTD, Jinan 250101, P. R. China

[4] Sinopec Qingdao Refining & Chemical CO., LTD, Qingdao 266500, P. R. China

*Corresponding authors

Email: wumb@upc.edu.cn, xin.tu@liv.ac.uk, wuwt@upc.edu.cn

†Qi C., Bi Y. and Wang Y. contributed equally to this work.

Table of contents

1. In situ FTIR characterization of the catalyst surface under plasma discharge

2. Schematic diagram of experimental setup and plasma reactor

3. Supplementary characterization of fresh catalysts

4. Supplementary reaction performance

5. Characterization of spent catalysts

6. In situ FTIR characterization results

7. CO-DRIFTS characterization results

8. Total liquid selectivity using 10%Cu/UiO-66-NH_2 at different CH_4/CO_2 ratios

9. Supplementary tables

1. In situ FTIR characterization of the catalyst surface under plasma discharge

In situ Fourier-transform infrared (FTIR) spectroscopy was used to characterize the catalyst surface using a custom-designed integrated DBD/gas cell, enabling the determination of various surface reactions and key intermediate species within the plasma-catalytic reaction system. Firstly, the catalyst surface was pretreated with argon plasma for 30 min (Ar flow rate 100 mL/min, discharge power 15 W, temperature 25 °C). Afterward, the catalyst sample was exposed to a mixture of CO_2 and CH_4 for 30 min (total flow rate: 100 mL/min, CO_2/CH_4 = 1:1), and then to a mixture of CO_2, CH_4 and argon for 30 min at a lower flow rate to collect the background FTIR spectrum (total flow rate: 20 mL/min, $CO_2/CH_4/Ar$ = 1:1:2). Under the condition of continuous flow (total flow rate: 20 mL/min, $CO_2/CH_4/Ar$ = 1:1:2), the plasma was switched on for 15 min, and the FTIR spectra were collected every 3 min for 15 min.

To understand the formation of surface intermediates species under CO_2 plasma exposure, the following test was conducted. The catalyst surface was firstly pretreated with argon plasma for 30 min (Ar flow rate 100 mL/min, discharge power 15 W, temperature 25 °C). After Ar plasma pretreatment, the catalyst sample was exposed to pure CO_2 (total flow rate: 100 mL/min) for 30 min and then to a mixture of argon and CO_2 at a lower flow rate for 30 min (total flow rate: 20 mL/min, CO_2/Ar = 1:1). Under the condition of continuous flow (total flow rate: 20 mL/min, CO_2/Ar = 1:1), the plasma was switched on for 15 min, and the FTIR spectra were collected every 3 min for 15 min.

To understand the reaction between CO_2 and CH_4 on the catalyst surface, CO_2 was

firstly absorbed onto the catalyst surface, and then the plasma was switched on under CH_4 atmosphere. The test procedure was as follows. After Ar plasma pretreatment, the catalyst was exposed to CO_2 (total flow rate: 100 mL/min) for 30 min and then to a mixture of argon and CH_4 at a lower flow rate for 30 min (total flow rate: 20 mL/min, CH_4/Ar = 1:1). Under the condition of continuous flow (total flow rate: 20 mL/min, CH_4/Ar = 1:1), the plasma was switched on for 15 min, and the FTIR spectra were collected every 3 min for 15 min to monitor the evolution of the reaction intermediates and surface species.

2. Schematic diagram of experimental setup and plasma reactor

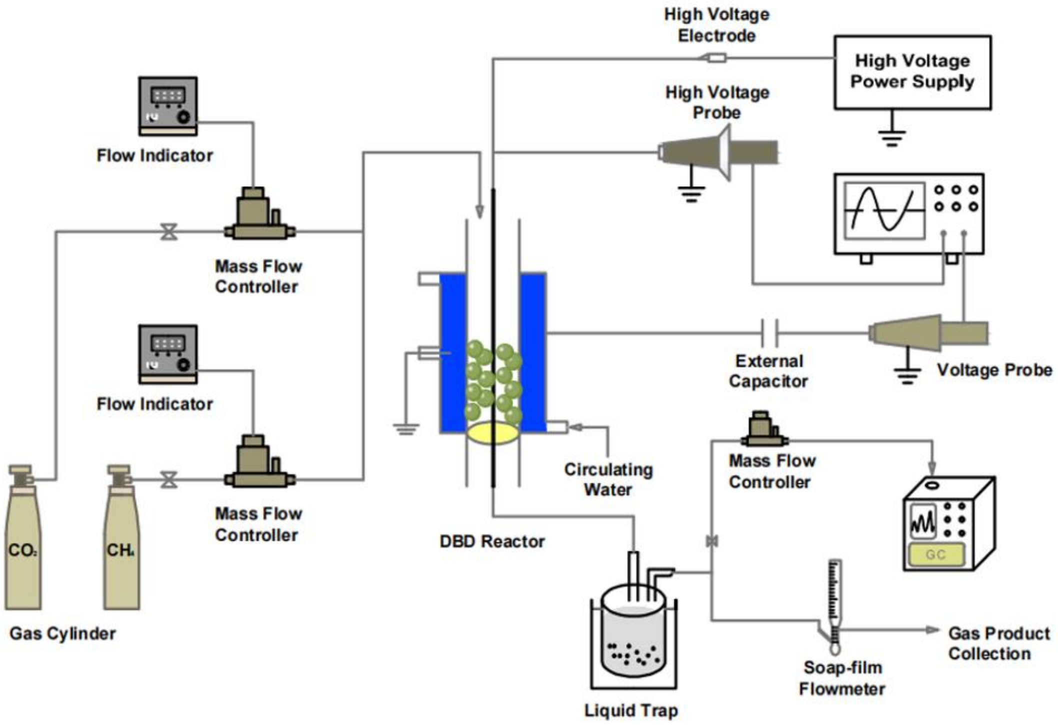

Scheme S1. Schematic diagram of the experimental setup.

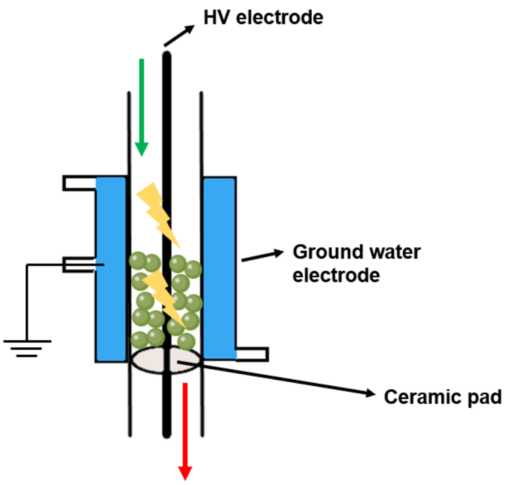

Scheme S2. Schematic diagram of the DBD reactor.

3. Supplementary characterization of fresh catalysts

Characterization of UiO-66: Catalyst characterization confirmed the successful synthesis of UiO-66 with an octahedral structure and a particle size of ~250 nm.

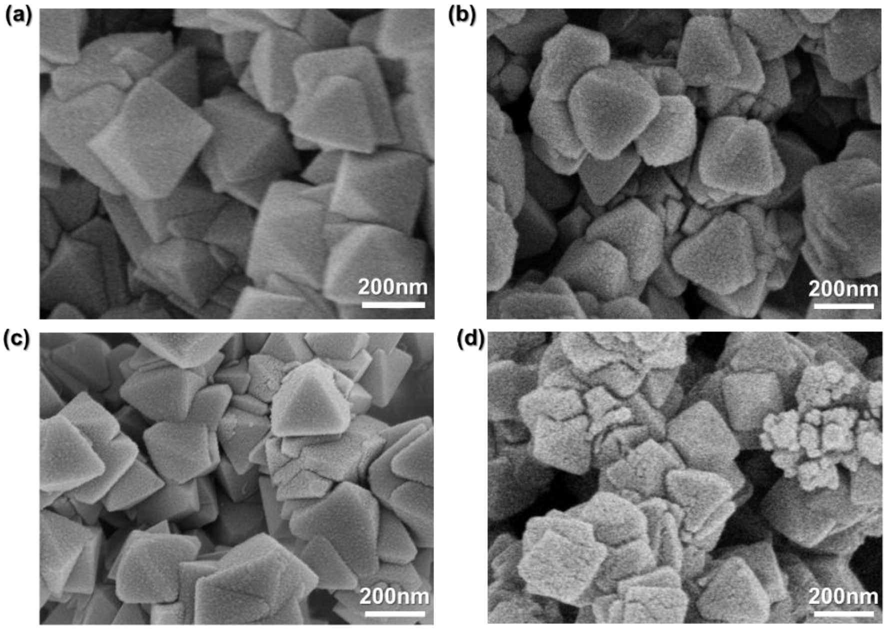

Figure S1. Scanning electron microscope (SEM) images of fresh catalysts (a) UiO-66-NH_2. (b) 5%Cu/UiO-66-NH_2. (c) 10%Cu/UiO-66-NH_2. (d) 15%Cu/UiO-66-NH_2.

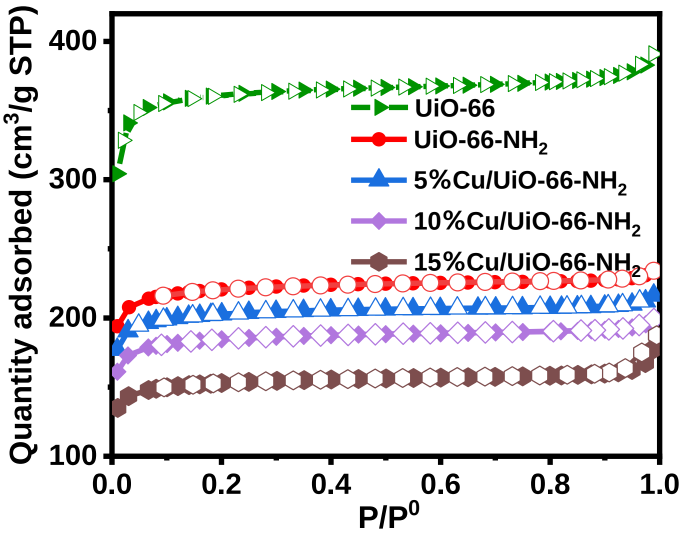

Figure S2. N_2 adsorption-desorption isotherms of fresh catalysts.

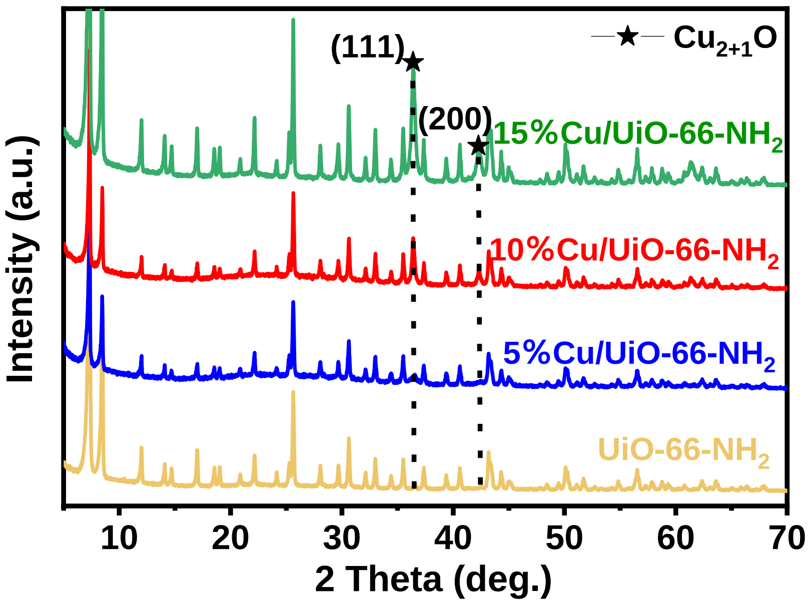

Figure S3. XRD patterns of UiO-66-NH_2 and XCu/UiO-66-NH_2.

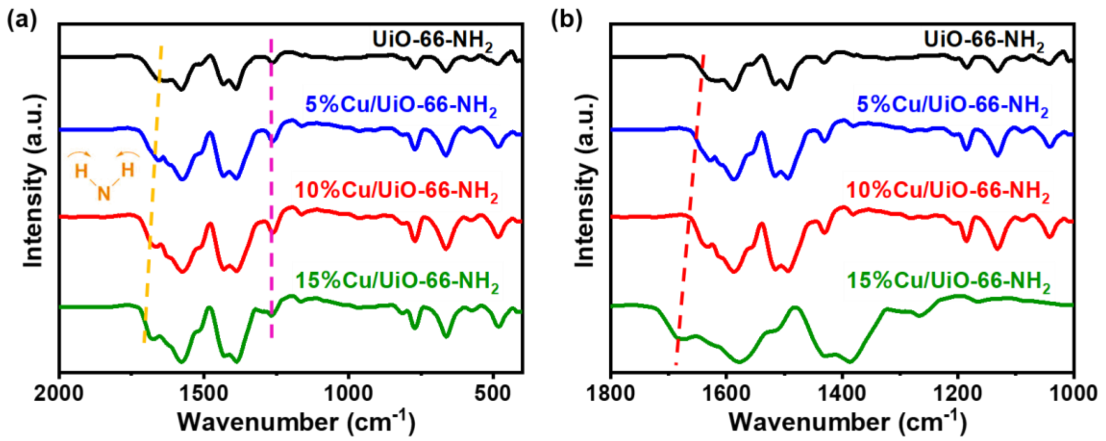

Figure S4. FTIR spectra of UiO-66-NH_2 and X%Cu/UiO-66-NH_2 (a) 2000-400 cm^{-1}. (b) 1800 cm^{-1} - 1000 cm^{-1}.

In Figure S4, the -NH_2 peak of UiO-66-NH_2 originally located at ~1620 cm^{-1} shifts to 1650 cm^{-1}, 1660 cm^{-1} and 1670 cm^{-1} with increasing Cu loading from 0% to 15%, (Figure S4a and S4b). These shifts indicate a coordination interaction between Cu and the amino group on the surface of the catalyst.

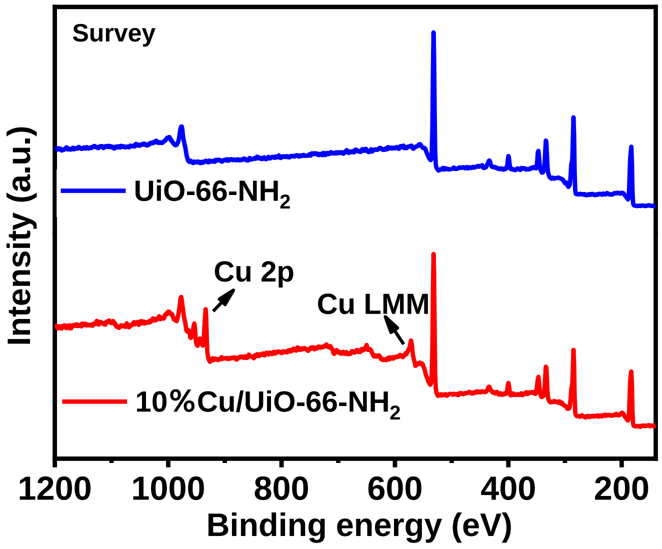

Figure S5. XPS spectra of UiO-66-NH_2 and 10%Cu/UiO-66-NH_2.

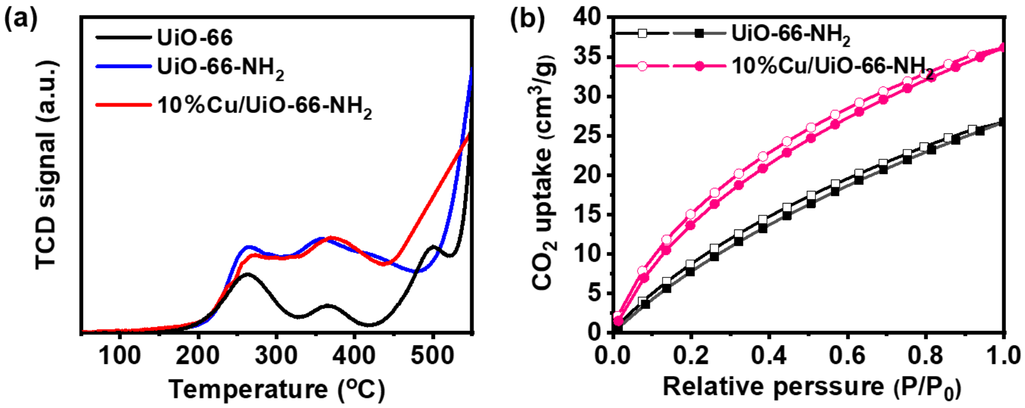

Figure S6. (a) CO_2-temperature programmed desorption of UiO-66, UiO-66-NH_2 and 10%Cu/UiO-66-NH_2. (b) Adsorption and desorption isotherms of CO_2 on UiO-66-NH_2 and 10%Cu/UiO-66-NH_2.

4. Supplementary reaction performance

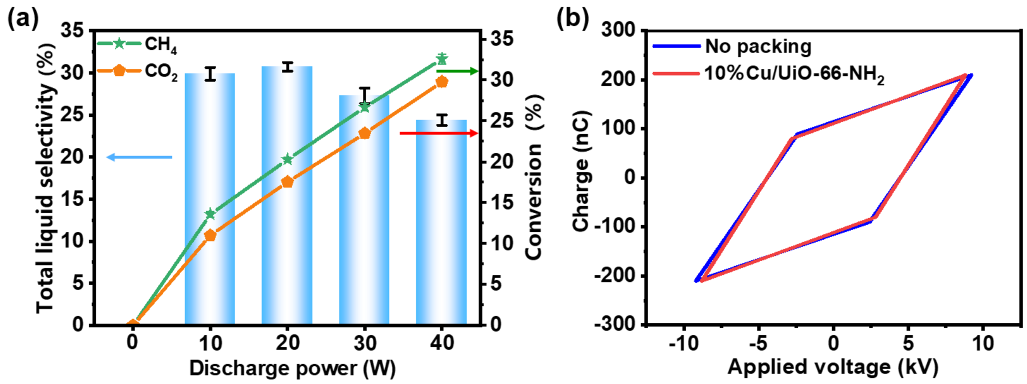

Figure S7. (a) Effect of discharge power on the conversion of CH_4 and CO_2 and the total selectivity of liquid products. (b) Lissajous figures (total flow rate = 50 mL/min, CH_4/CO_2 = 1:1, discharge power = 20 W).

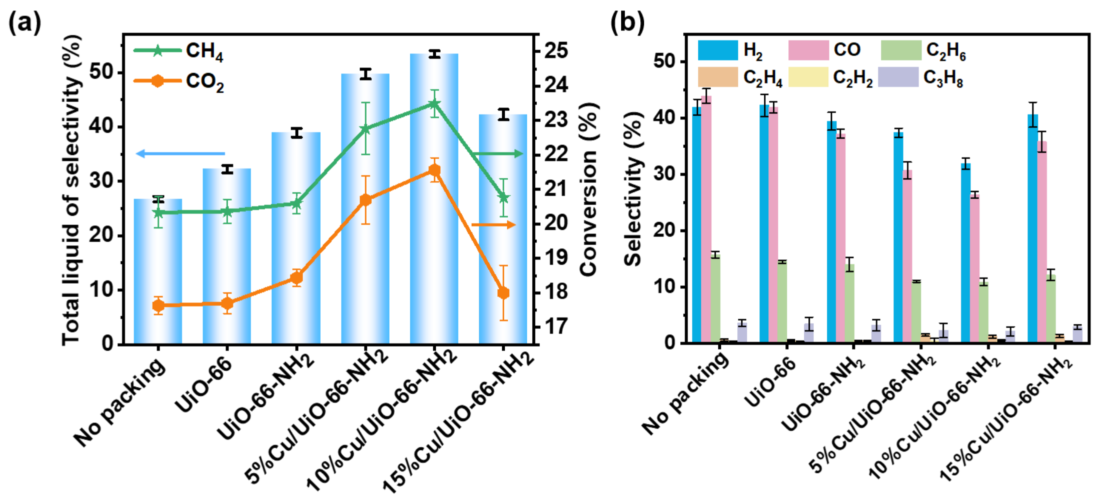

Figure S8. (a) Conversion of CH_4 and CO_2 and total liquid of selectivity. (b) Selectivity of gaseous products without packing, with UiO-66, UiO-66-NH_2, 10%Cu/UiO-66-NH_2 (total gas flow rate = 50 mL/min, CH_4/CO_2 = 1:1, discharge power = 20 W)

5. Characterization of spent catalysts

Thermogravimetric analysis (TGA) was used to quantitatively determine carbon deposition on the catalysts. Since UiO-66-NH_2 exhibits limited thermal stability and its framework tends to decompose at elevated temperatures, TGA measurements were conducted in both N_2 and air atmospheres with four repeated experiments (80 min each) on both fresh and spent 10%Cu/UiO-66-NH_2 catalysts.

In the N_2 atmosphere, weight loss began around 400 °C, mainly due to the collapse of the UiO-66-NH_2 framework. In contrast, in the air atmosphere, weight loss occurred between 300 °C and 400 °C, likely due to catalyst decomposition. The weight loss of the spent catalyst included both catalyst degradation and carbon deposition. After subtracting the weight loss attributed to water desorption at 100 °C, the weight loss for fresh and spent catalysts in the N_2 atmosphere was 21.1% and 24.3% between 30 and 600 °C, respectively. Similarly, the weight loss of the catalysts in air was 30.5% and 39.8%. These data indicate a carbon deposition of 6.6%, corresponding to an actual carbon deposition selectivity of 3.7%.

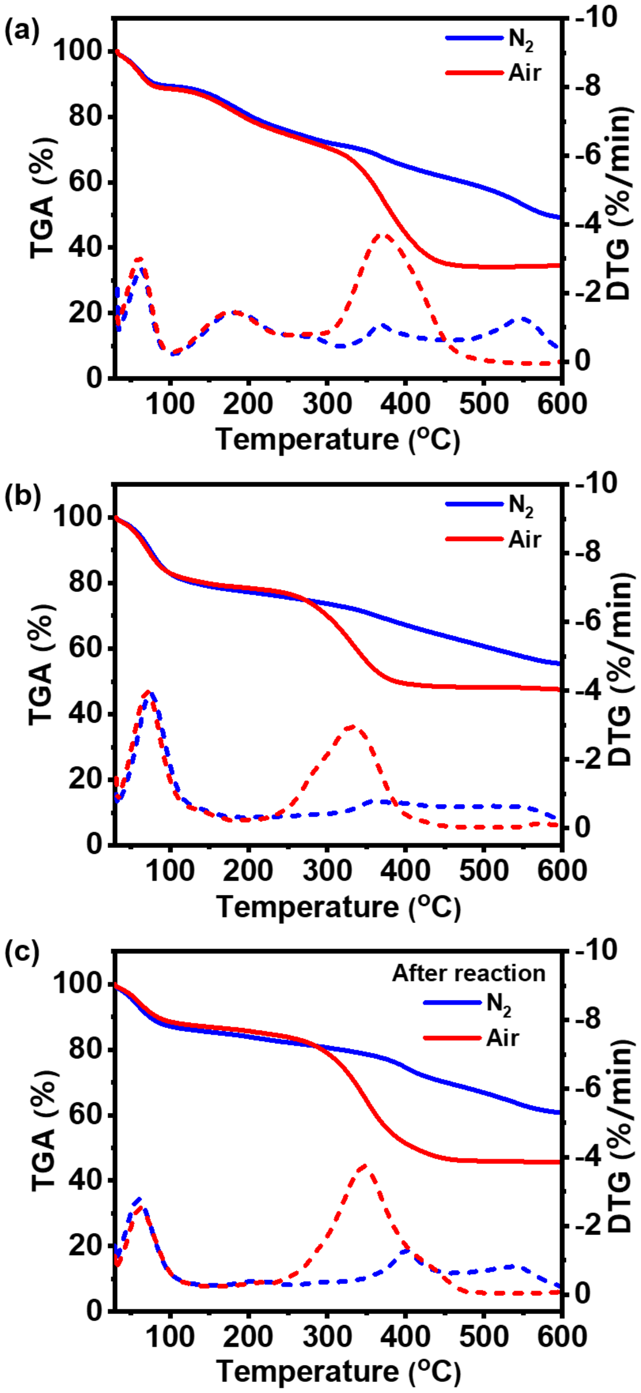

Figure S9. TGA-DTG curves of (a) UiO-66-NH_2. (b) Fresh 10%Cu/UiO-66-NH_2. (c) spent 10%Cu/UiO-66-NH_2.

Characterization of catalysts after reaction: Examination of the spent 10%Cu/UiO-66-NH_2 catalyst (XRD, FTIR, and SEM) revealed that its crystallinity and structure remained intact after the plasma reaction (Figure S10 a-c). This suggests that the catalyst exhibited remarkable stability during the plasma catalytic reaction.

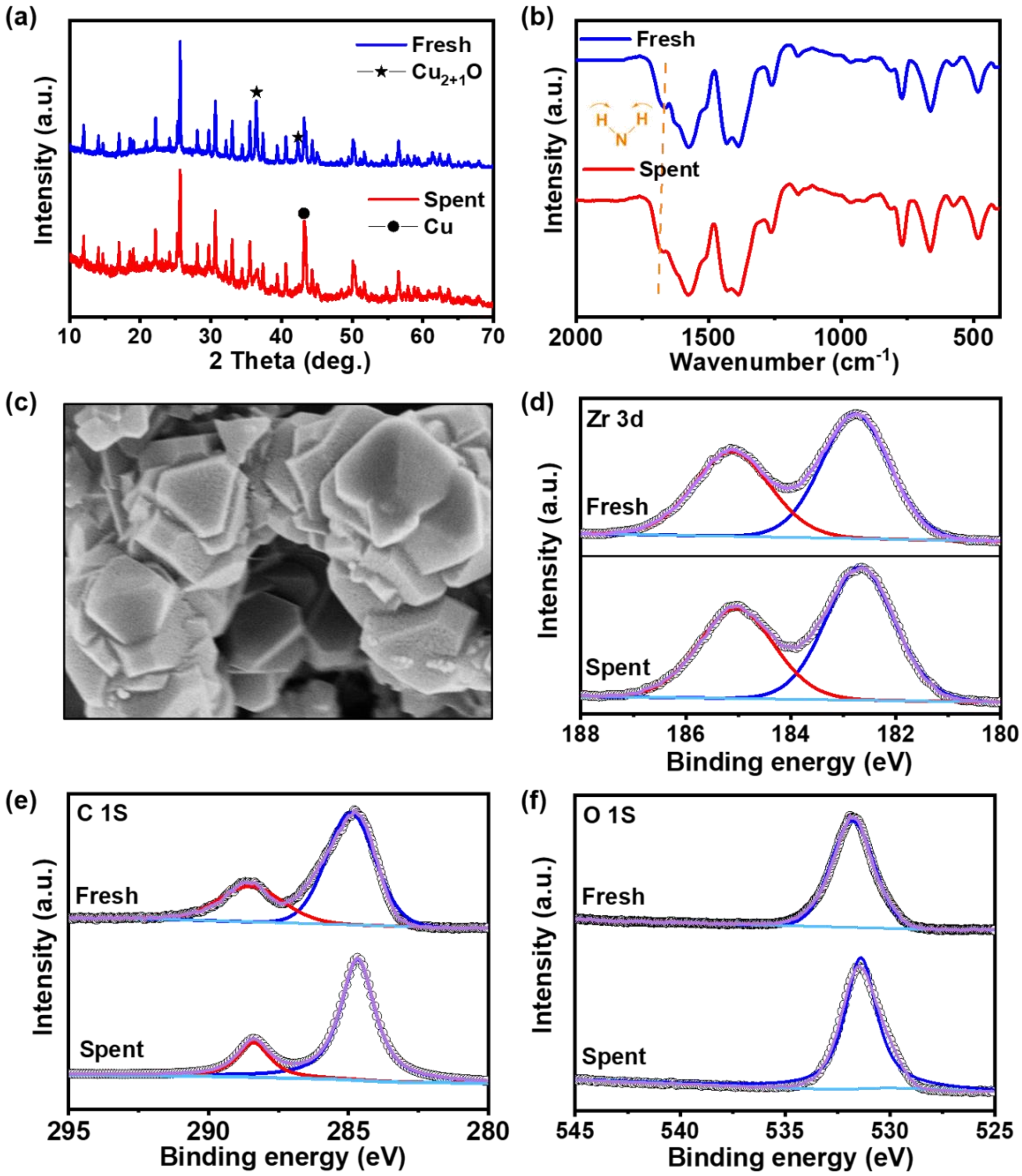

Figure S10. Characterization of fresh and spent 10%Cu/UiO-66-NH_2 (a) XRD patterns. (b) FTIR spectra. (c) SEM images. XPS spectra of (d) Zr 3d. (e) C 1s. (f) O 1s (total flow rate = 50 mL/min, CH_4/CO_2 = 1:1, discharge power = 20 W, reaction time 20 min).

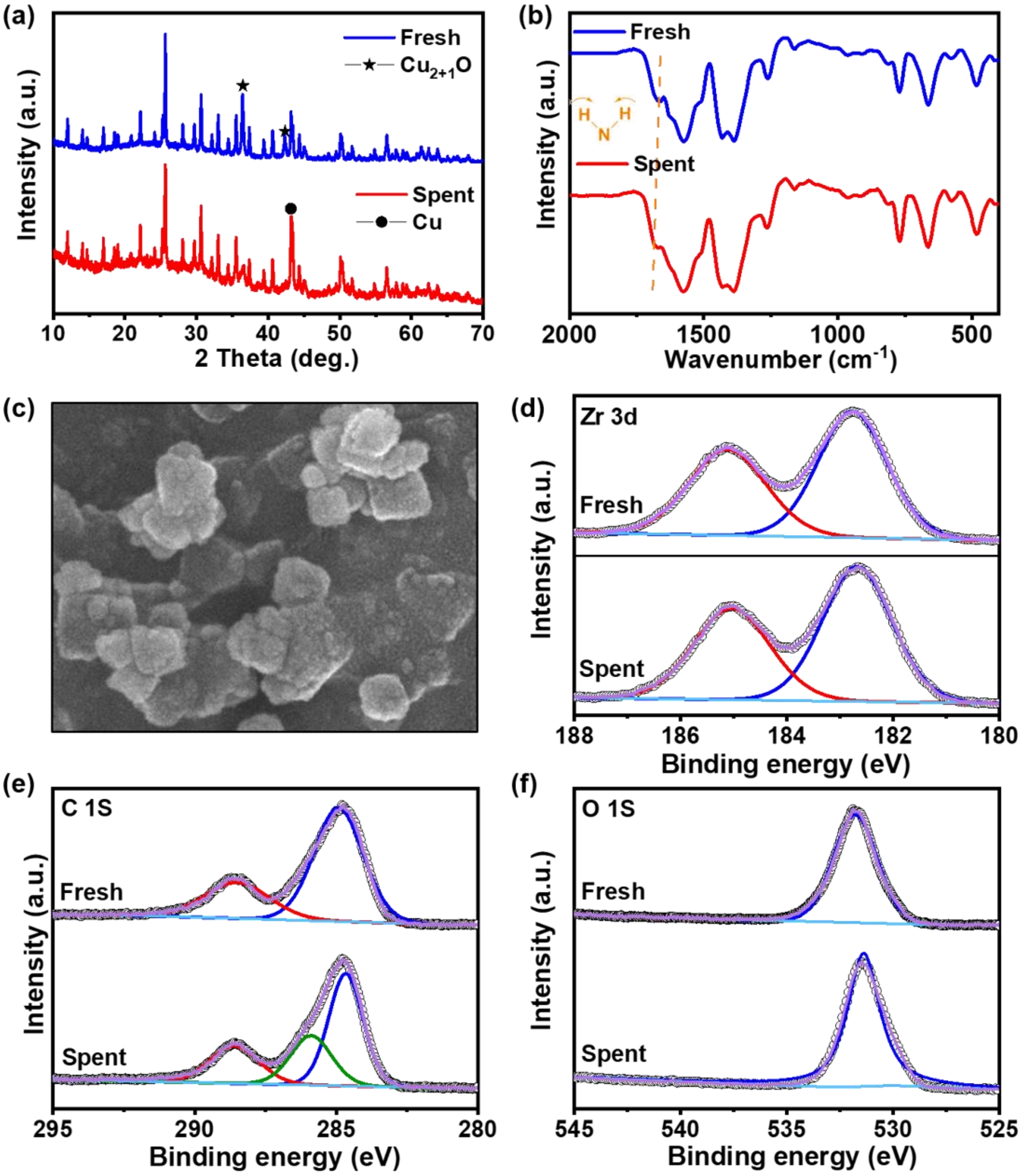

Figure S11. Characterization of fresh and spent 10%Cu/UiO-66-NH_2 (a) XRD patterns. (b) FTIR spectra. (c) SEM images. XPS spectra of (d) Zr 3d (e) C 1s (f) O 1s (Reaction time 240 min).

6. In situ FTIR characterization results

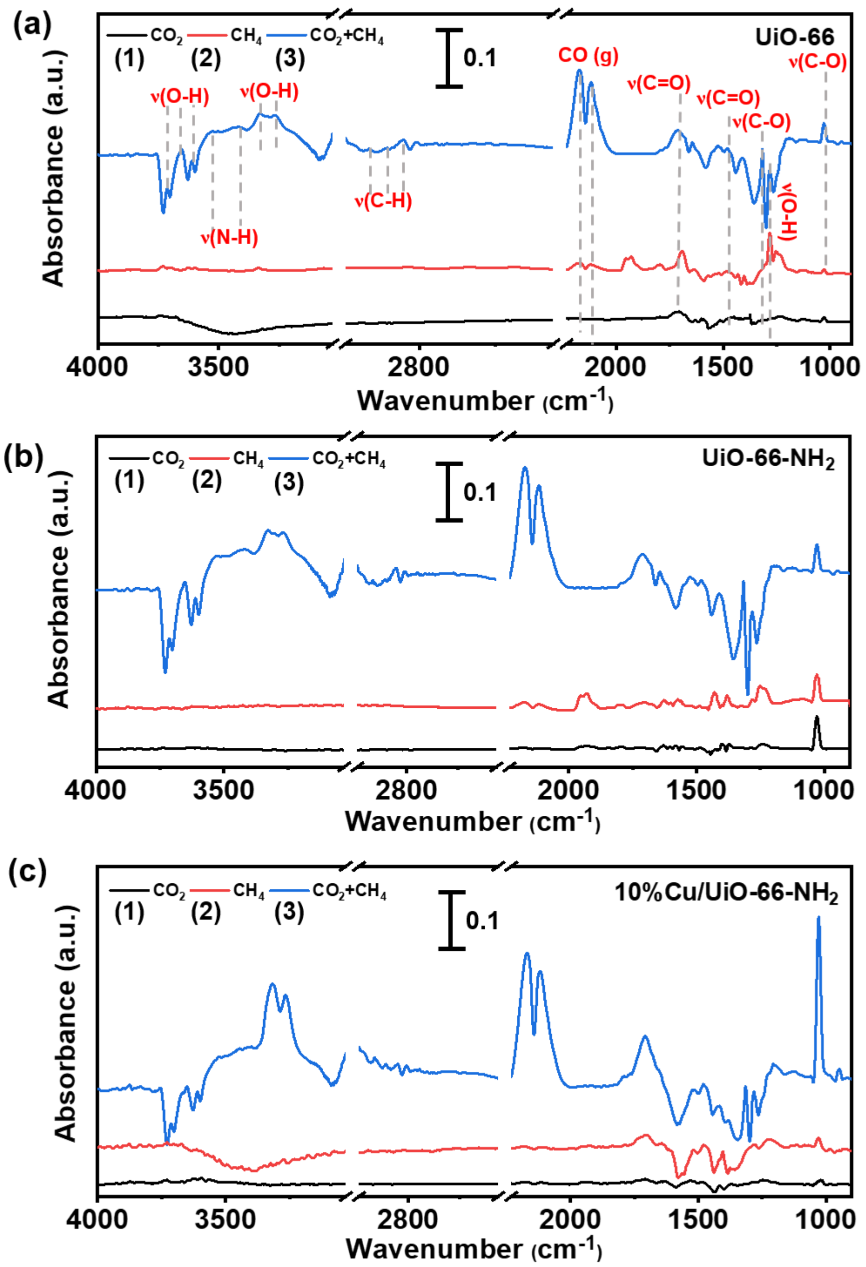

Figure S12. In situ transmission FTIR spectra of different catalysts during plasma catalytic DRM reaction under continuous flow and different atmospheres (a) UiO-66. (b) UiO-66-NH_2. (c) 10%Cu/UiO-66-NH_2 (discharge power = 15 W, Conditon (1): Pure CO_2 atmosphere; Condition (2): Catalyst surface initially adsorbed with CO_2 followed by reaction in a CH_4 atmosphere, condition; (3): Reaction under a CO_2 and CH_4 atmosphere with a CH_4/CO_2 ratio of 1:1)

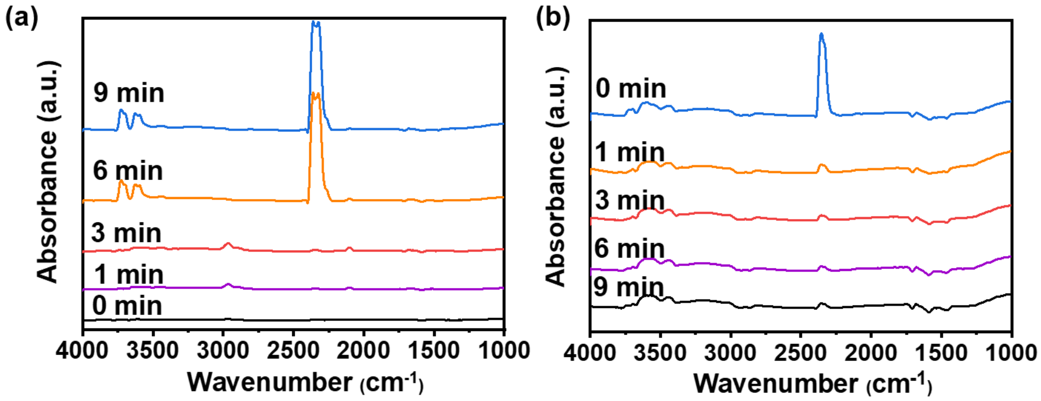

Figure S13. (a) DRIFT spectra of 10%Cu/UiO-66-NH_2 during CO_2 adsorption. (b) DRIFT spectra of 10%Cu/UiO-66-NH_2 after CO_2 adsorption with subsequent N_2 flush (CO_2 flow rate = 20 mL/min, temperature = 35 °C)

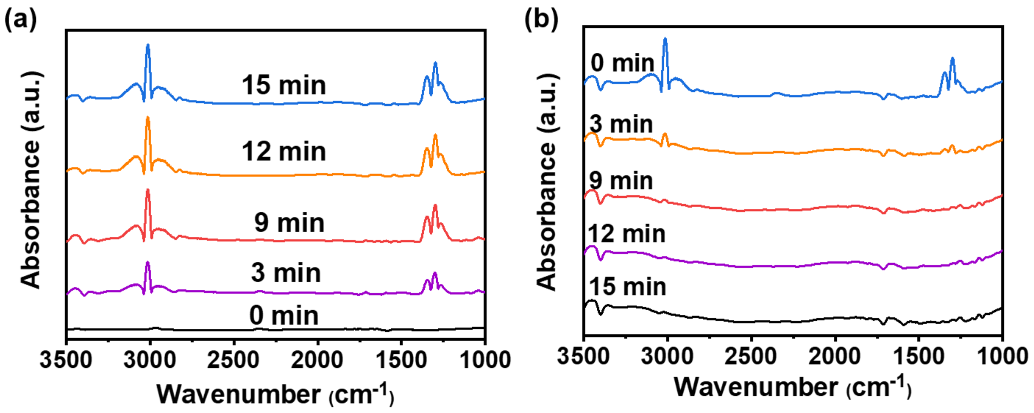

Figure S14. (a) DRIFT spectra of 10%Cu/UiO-66-NH_2 during CH_4 adsorption. (b) DRIFT spectra of 10%Cu/UiO-66-NH_2 after CH_4 adsorption with subsequent N_2 flush (CH_4 flow rate = 20 mL/min, temperature = 35 °C)

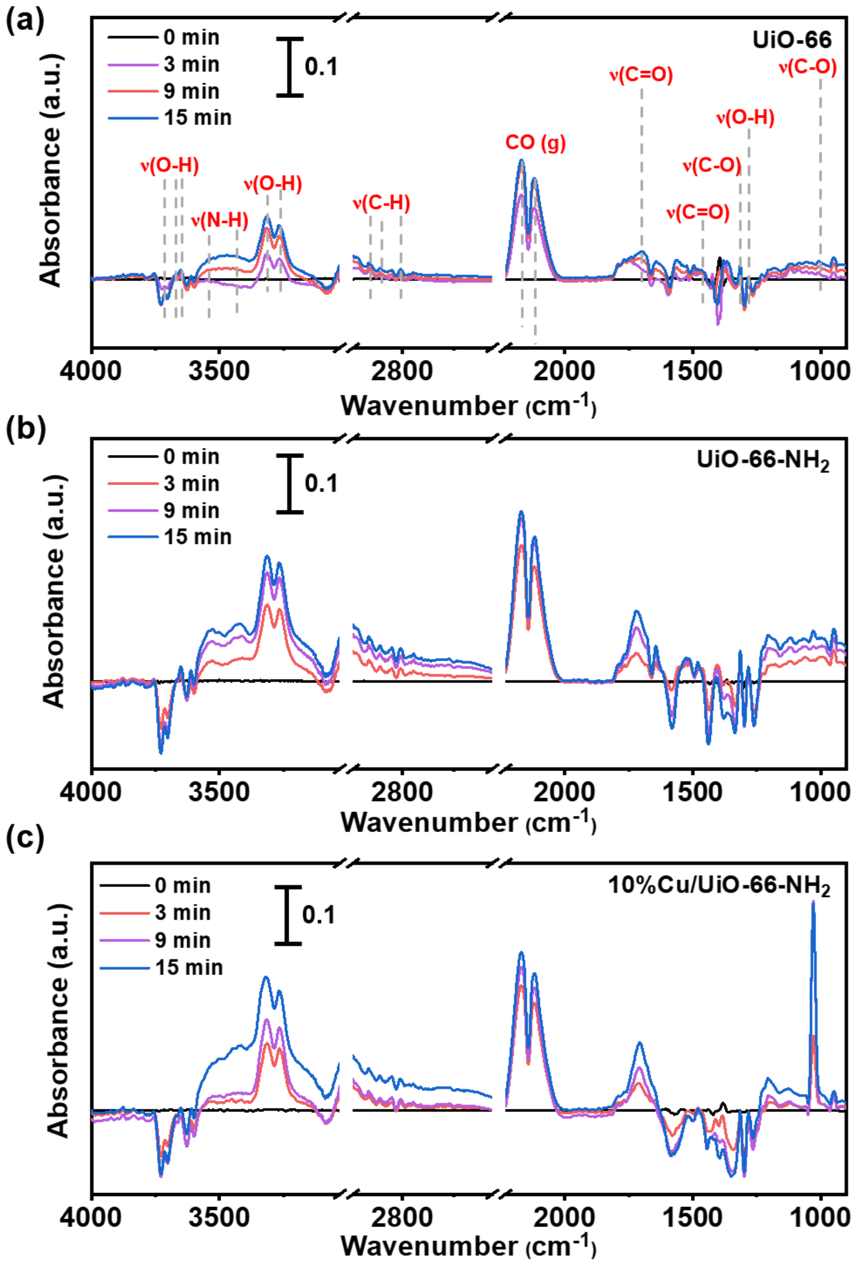

Figure S15. In situ transmission FTIR spectra of different catalysts during plasma-catalytic DRM reaction (a) UiO-66. (b) UiO-66-NH_2. (c) 10%Cu/UiO-66-NH_2 (CO_2/CH_4/Ar = 1:1:2, total gas flow rate = 20 mL/min, discharge power = 15 W)

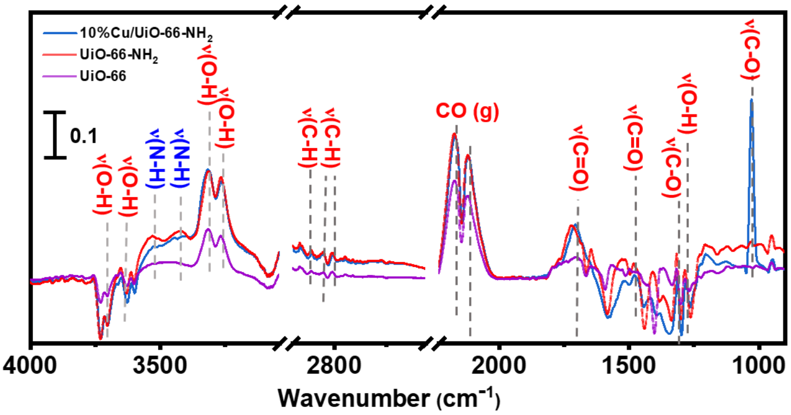

Figure S16. In situ transmission FTIR spectra of different catalysts during plasma-catalytic DRM reaction (at 15 min) (CO_2/CH_4/Ar = 1:1:2, total gas flow rate = 20 mL/min , discharge power = 15 W).

7. CO-DRIFTS characterization results

Both 10% Cu/UiO-66-NH_2 and UiO-66-NH_2 exhibited strong physical adsorption of CO(g), consistent with the results of in situ transmission FTIR characterization. The desorption profiles of CO showed that the physical absorption peaks of CO(g) at 2170 cm^{-1} of the two catalysts gradually disappeared with N_2 purging. Notably, the absorption peak of 10% Cu/UiO-66-NH_2 at 2119 cm^{-1} gradually shifted towards low wavenumber with purge time. Eventually, it stabilized at 2110 cm^{-1}, corresponding to the linear adsorption of Cu^+-CO. In contrast, the absorption peak of UiO-66-NH_2 at 2119 cm^{-1} completely disappeared, indicating the superior chemical adsorption capacity of Cu for *CO.

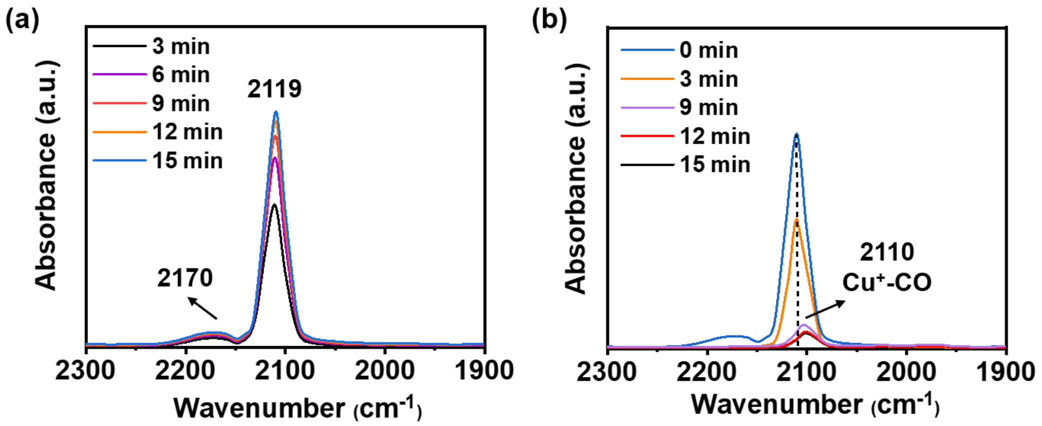

Figure S17. (a) DRIFT spectra of 10%Cu/UiO-66-NH_2 during CO adsorption. (b) DRIFT spectra of 10%Cu/UiO-66-NH_2 after CO adsorption with subsequent N_2 flush (5% CO/Ar gas flow rate = 20 mL/min).

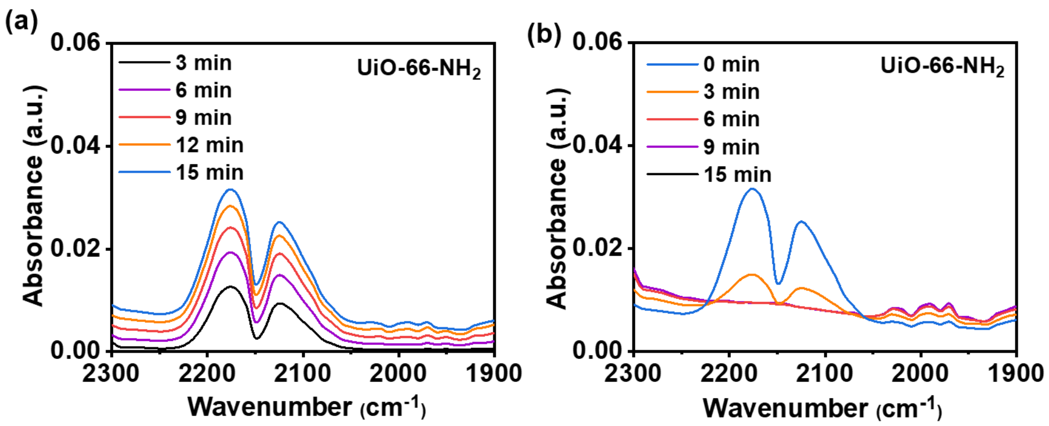

Figure S18. (a) DRIFT spectra of UiO-66-NH_2 during CO adsorption. (b) DRIFT spectra of UiO-66-NH_2 after CO adsorption with Ar flush (5% CO/Ar gas flow rate = 20 mL/min).

8. Total liquid selectivity using 10%Cu/UiO-66-NH_2 at different CH_4/CO_2 ratios

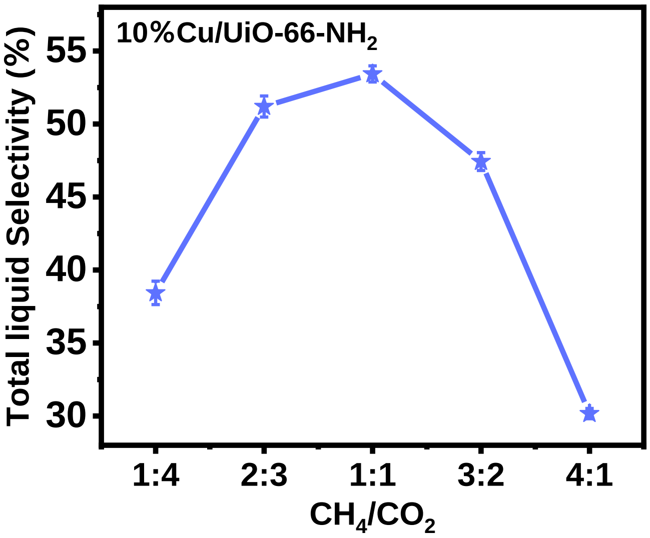

Figure S19. Selectivity of total liquid products using 10%Cu/UiO-66-NH_2 at different CH_4/CO_2 ratios (total flow rate = 50 mL/min, discharge power = 20 W)

9. Supplementary tables

Table S1. Inductively coupled plasma optical emission spectrometry (ICP-OES) analysis of 5%Cu/UiO-66-NH_2, 10%Cu/UiO-66-NH_2 and 15%Cu/UiO-66-NH_2.

Samples	Cu content (wt.%)
5%Cu/UiO-66-NH_2	4.55
10%Cu/UiO-66-NH_2	8.95
15%Cu/UiO-66-NH_2.	13.23

Table S2. S_{BET}, pore volume and pore diameter of fresh catalysts.

Samples	S_{BET}[a] (m^2/g)	Pore volume[b] (cm^3/g)	Pore diameter[c] (nm)
UiO-66	1111	0.61	2.18
UiO-66-NH_2	872	0.36	3.42
5%Cu/UiO-66-NH_2	803	0.34	3.68
10%Cu/UiO-66-NH_2	723	0.31	3.84
15%Cu/UiO-66-NH_2.	603	0.29	7.50

[a]The Brunauer-Emmett-Teller (BET) method was used to determine the specific surface area of samples.

[b]The single point method (at $p/p^0 = 0.99$) was used to determine the total pore volume.

[c]The BJH method was used to determine the average pore diameter.

Table S3. Effect of catalysts on the conversion and total liquid selectivity.

Sample	CO_2 (%)	CH_4 (%)	Total liquid selectivity (%)
No packing	17.6	20.3	30.7
UiO-66	17.7	20.4	32.2
UiO-66-NH_2	18.4	20.6	38.6
10%Cu/UiO-66-NH_2	21.6	23.5	53.4

Table S4. Effect of catalysts on the selectivity of gas products.

Sample	H_2 (%)	CO (%)	C_2H_6 (%)	C_2H_4 (%)	C_2H_2 (%)	C_3H_8 (%)
No packing	42.2	44.0	15.8	0.5	0.3	3.7
UiO-66	42.3	42.0	14.5	0.5	0.4	3.5
UiO-66-NH_2	39.5	37.2	14.0	0.5	0.4	3.2
10%Cu/UiO-66-NH_2	32.0	26.5	10.9	1.4	0.5	2.2

Table S5. Effect of catalysts on the selectivity of liquid products.

Samples	CH_3OH (%)	C_2H_5O (%)	C_3H_6O (%)	CH_3COOH (%)	CH_3CHO (%)
No packing	15.	9.5	4.5	0.7	0
UiO-66	17.1	9.8	4.6	0.6	0
UiO-66-NH_2	20.9	10.9	5.2	1.6	0
10%Cu/UiO-66-NH_2	20.9	18.4	8.6	3.3	2.3

Table S6. Conversion and total liquid selectivity at different CH_4/CO_2 ratios using 10%Cu/UiO-66-NH_2 (total gas flow rate = 50 mL/min, discharge power = 20 W).

CH_4/CO_2	CH_4 (%)	CO_2 (%)	Total liquid selectivity (%)
1:4	33.2	10.2	38.5
2:3	28.3	16.3	51.1
1:1	23.5	21.6	53.4
3:2	21.0	24.2	47.3
4:1	15.4	29.0	30.2

Table S7. Gas selectivity at different CH_4/CO_2 ratios using 10%Cu/UiO-66-NH_2 (total gas flow rate = 50 mL/min, discharge power = 20 W).

CH_4/CO_2	H_2 (%)	CO (%)	C_2H_6 (%)	C_2H_4 (%)	C_2H_2 (%)	C_3H_8 (%)
1:4	24.0	44.7	7.2	1.1	0.2	1.6
2:3	31.1	29.6	10.2	1.1	0.3	2.1
1:1	32.0	26.8	10.9	1.4	0.5	2.0
3:2	36.0	26.3	14.8	1.6	0.8	3.2
4:1	48.0	19.3	27.2	3.6	1.8	6.1

Table S8. Summary of conversions and liquid selectivities reported in the literature.

Reference	CO_2 (%)	CH_4 (%)	Total liquid selectivity (%)	C_1 liquid selectivity (%)	C_2 liquid selectivity (%)
1	15.4	18.3	59.1	~10.0	~49.2
2	29.6	43.2	~40.0	~20.5	~17.9
3	29.0	~32.0	60.0	~40.0	~20.0
4	~17	~7	32.0	20.0	12.0
5	-	18.4	~12	~12.0	-
6	21.0	13.2	1.8	-	-
7	25.0	43.0	~16.2	~6.5	~9.7
8	12.0	8.0	37.0	-	-
9	26.8	47.5	~4.4	~2.3	~1.1
10	12.0	6.0	~2.0	-	-
This work	21.6	23.5	53.4	21.0	32.4

Table S9. Species identified in the plasma-catalytic DRM reaction over 10%Cu/UiO-66-NH_2 by in situ transmission FTIR.

Species	Assignment	Wavenumber (cm^{-1})	Reference
Alcohols	C-O stretching vibration	1028	11,12
COOH	OH stretching vibration	1282	13, 14, 15
COOH	C-O stretching vibration	1314, 1315	13, 14, 15
COOH	C=O stretching vibration	1479, 1495	13, 14, 15
NH_2COOH	C=O stretching vibration	1716	16,17, 18
CO (g)	Gas phase	2119, 2170	13, 19, 20
CH_3O	C-H stretching vibration	2807	21
CH_3	C-H stretching vibration	2840, 2888	22, 23
CH_2	C-H stretching vibration	2922	24
NH_2	N-H stretching vibration	3532, 3419	25, 26
Surface OH	OH stretching	3316, 3267	27
Isolated OH	OH stretching	3611, 3650, 3711	12, 21

Table S10. Liquid selectivity at different CH_4/CO_2 ratios using 10%Cu/UiO-66-NH_2 (total gas flow rate = 50 mL/min, discharge power = 20 W).

CH_4/CO_2	CH_3OH (%)	C_2H_5O (%)	C_3H_6O (%)	CH_3COOH (%)	CH_3CHO (%)
1:4	19.7	9.6	3.8	2.5	2.8
2:3	23.2	14.1	5.8	2.7	5.2
1:1	21.2	18.3	8.2	3.2	2.4
3:2	16.5	13.5	9.7	1.4	6.2
4:1	9.7	12.2	4.5	0.8	3.1

References

(1) Wang, L.; Yi, Y.; Wu, C.; Guo, H.; Tu, X. One-Step Reforming of CO_2 and CH_4 into High-Value Liquid Chemicals and Fuels at Room Temperature by Plasma-Driven Catalysis. *Angew. Chem. Int. Ed.* **2017**, *56*, 13679-13683.

(2) Li, D.; Rohani, V.; Fabry, F.; Parakkulam Ramaswamy, A.; Sennour, M.; Fulcheri, L. Direct conversion of CO_2 and CH_4 into liquid chemicals by plasma-catalysis. *Appl. Catal. B-Environ.* **2020**, *261*, 118228.

(3) Wang, Y.; Chen, Y.; Harding, J.; He, H.; Bogaerts, A.; Tu, X. Catalyst-free single-step plasma reforming of CH_4 and CO_2 to higher value oxygenates under ambient conditions. *Chem. Eng. J.* **2022**, *450*, 137860.

(4) Wang, L.; Wang, Y.; Fan, L.; Xu, H.; Liu, B.; Zhang, J.; Zhu, Y.; Tu, X. Direct conversion of CH_4 and CO_2 to alcohols using plasma catalysis over $Cu/Al(OH)_3$ catalysts. *Chem. Eng. J.* **2023**, *466*, 143347.

(5) Gorky, F.; Nambo, A.; Carreon, M. Cold plasma-Metal Organic Framework (MOF)-177 breathable system for atmospheric remediation. *J. CO_2 Util.* **2021**, *51*, 101642.

(6) Bouchoul, N.; Fourré, E.; Tatibouët, J.; Duarte, A.; Tanchoux, N.; Batiot-Dupeyrat, C. Structural modifications of calcium based catalysts by non-thermal plasma in the CO_2 reforming of CH_4 and the influence of water. *J. CO_2 Util.* **2020**, *35*, 79-89.

(7) Rahmani, A.; Nikravech, M. Impact of Argon in Reforming of ($CH_4 + CO_2$) in Surface Dielectric Barrier Discharge Reactor to Produce Syngas and Liquid Fuels. *Plasma Chem. Plasma P.* **2018**, *38*, 517-534.

(8) Scapinello, M.; Martini, L. M.; Tosi, P. CO_2 Hydrogenation by CH_4 in a Dielectric Barrier Discharge: Catalytic Effects of Nickel and Copper. *Plasma Process. Polym.* **2014**, *11*, 624-628.

(9) Krawczyk, K.; Młotek, M.; Ulejczyk, B.; Schmidt-Szałowski, K. Methane conversion with carbon dioxide in plasma-catalytic system. *Fuel* **2014**, *117*, 608-617.

(10) Martini, L. M.; Dilecce, G.; Guella, G.; Maranzana, A.; Tonachini, G.; Tosi, P. Oxidation of CH_4 by CO_2 in a Dielectric Barrier Discharge. *Chem. Phys. Lett.* **2014**, *593*, 55-60.

(11) Shi, J.; Mahr, C.; Murshed, M.; Gesing, T.; Rosenauer, A.; Baumer, M.; Wittstock, A. Steam reforming of methanol over oxide decorated nanoporous gold catalysts: a combined in situ FTIR and flow reactor study. *Phys. Chem. Chem. Phys.* **2017**, *19*, 8880-8888.

(12) Chen, T.; Feng, Z.; Wu, G.; Shi, J.; Ma, G.; Ying, P.; Li, C. Mechanistic Studies of Photocatalytic Reaction of Methanol for Hydrogen Production on Pt/TiO_2 by in situ Fourier Transform IR and Time-Resolved IR Spectroscopy. *J. Phys. Chem. C.* **2007**, *111*, 8005-8014.

(13) Zheng, J.; Wang, C.; Chu, W.; Zhou, Y.; Köhler, K. CO_2 Methanation over Supported Ru/Al_2O_3 Catalysts: Mechanistic Studies byIn situInfrared Spectroscopy. *Chem. Select.* **2016**, *1*, 3197-3203.

(14) Chen, H.; Mu, Y.; Shao, Y.; Chansai, S.; Xiang, H.; Jiao, Y.; Hardacre, C.; Fan, X. Nonthermal plasma NTP activated metal-organic frameworks MOFs catalyst for catalytic CO_2 hydrogenation. *AIChE J.* **2019**, *66*, 16583.

(15) Liu, P.; Huang, Z.; Gao, X.; Hong, X.; Zhu, J.; Wang, G.; Wu, Y.; Zeng, J.; Zheng, X. Synergy between Palladium Single Atoms and Nanoparticles via Hydrogen Spillover for Enhancing CO_2 Photoreduction to CH_4. *Adv. Mater.* **2022**, *34*, e2200057.

(16) Danon, A.; Stair, P.; Weitz, E. FTIR Study of CO_2 Adsorption on Amine-Grafted SBA-15: Elucidation of Adsorbed Species. *J. Phys. Chem. C.* **2011**, *115*, 11118-11128.

(17) Yang, S.; Pang, S.; Sulmonetti, T.; Su, W.; Lee, J.; Hwang, B.; Jones, C. Synergy between Ceria Oxygen Vacancies and Cu Nanoparticles Facilitates the Catalytic Conversion of CO_2 to CO under Mild Conditions. *ACS Catal.* **2018**, *8*, 12056-12066.

(18) Bacsik, Z.; Ahlsten, N.; Ziadi, A.; Zhao, G.; Garcia-Bennett, A. E.; Martin-Matute, B.; Hedin, N. Mechanisms and kinetics for sorption of CO_2 on bicontinuous mesoporous silica modified with n-propylamine. *Langmuir* **2011**, *27*, 11118-28.

(19) Vakili, R.; Gholami, R.; C., S.; Chansai, S.; Chen, H.; Holmes, S.; Jiao, Y.; Hardacre, C.; Fan, X. Plasma-assisted Catalytic Dry Reforming of Methane (DRM) over Metalorganic Frameworks (MOFs)-based Catalysts. *Appl. Catal. B-Environ.* **2020**, *260*, 118195.

(20) Bobadilla, L.; Garcilaso, V.; Centeno, M.; Odriozola, J. Monitoring the Reaction Mechanism in Model Biogas Reforming by In Situ Transient and Steady-State DRIFTS Measurements. *ChemSusChem* **2017**, *10*, 1193-1201.

(21) Eckle, S.; Anfang, H.; Behm, R. What drives the selectivity for CO methanation in the methanation of CO_2-rich reformate gases on supported Ru catalysts? *Appl. Catal. A-Gen.* **2011**, *391*, 325-333.

(22) Sushkevich, V.; Palagin, D.; Ranocchiari, M.; Bokhoven, J. Selective anaerobic

oxidation ofmethane enables directsynthesis of methanol. *Science* **2017**, *356*, 523-527.

(23) Song, S.; Song, H.; Li, L.; Wang, S.; Chu, W.; Peng, K.; Meng, X.; Wang, Q.; Deng, B.; Liu, Q.; Wang, Z.; Weng, Y.; Hu, H.; Lin, H.; Kako, T. A Selective Au-ZnO/TiO_2 Hybrid Photocatalyst for Oxidative Coupling of Methane to Ethane with Dioxygen. *Nat. Catal.* **2021**, *4*, 1032-1042.

(24) Yu, J.; Zhai, Y.; Chuang, S. Water Enhancement in CO_2 Capture by Amines: An Insight into CO_2-H_2O Interactions on Amine Films and Sorbents. *Ind. Eng. Chem. Res.* **2018**, *57*, 4052-4062.

(25) Fan, Z.; Shi, J. W.; Gao, C.; Gao, G.; Wang, B.; Niu, C. Rationally Designed Porous MnOx-FeOx Nanoneedles for Low-Temperature Selective Catalytic Reduction of NOx by NH_3. *ACS Appl. Mater. Interfaces.* **2017**, *9*, 16117-16127.

(26) Wang, Q.; Xu, H.; Huang, W.; Pan, Z.; Zhou, H. Metal organic frameworks-assisted fabrication of CuO/Cu_2O for enhanced selective catalytic reduction of NO_x by NH_3 at low temperatures. *J. Haz. Mat.* **2019**, *364*, 499-508.

(27) Moon, J.; Cheng, Y.; Daemen, L.; Li, M.; Polo-Garzon, F.; Ramirez-Cuesta, A.; Wu, Z. Discriminating the Role of Surface Hydride and Hydroxyl for Acetylene Semihydrogenation over Ceria through In Situ Neutron and Infrared Spectroscopy. *ACS Catal.* **2020**, *10*, 5278-5287.
